# Sequence- and Structure-Based Functional Annotation and Assessment of Metabolic Transporters in* Aspergillus oryzae*: A Representative Case Study

**DOI:** 10.1155/2016/8124636

**Published:** 2016-05-04

**Authors:** Nachon Raethong, Jirasak Wong-ekkabut, Kobkul Laoteng, Wanwipa Vongsangnak

**Affiliations:** ^1^Department of Zoology, Faculty of Science, Kasetsart University, Bangkok 10900, Thailand; ^2^Department of Physics, Faculty of Science, Kasetsart University, Bangkok 10900, Thailand; ^3^Computational Biomodelling Laboratory for Agricultural Science and Technology (CBLAST), Faculty of Science, Kasetsart University, Bangkok 10900, Thailand; ^4^Center of Advanced Science in Industrial Technology, Faculty of Science, Kasetsart University, Bangkok 10900, Thailand; ^5^Food Biotechnology Research Unit, National Center for Genetic Engineering and Biotechnology (BIOTEC), National Science and Technology Development Agency (NSTDA), Pathum Thani 12120, Thailand

## Abstract

*Aspergillus oryzae* is widely used for the industrial production of enzymes. In* A. oryzae* metabolism, transporters appear to play crucial roles in controlling the flux of molecules for energy generation, nutrients delivery, and waste elimination in the cell. While the* A. oryzae *genome sequence is available, transporter annotation remains limited and thus the connectivity of metabolic networks is incomplete. In this study, we developed a metabolic annotation strategy to understand the relationship between the sequence, structure, and function for annotation of* A. oryzae* metabolic transporters. Sequence-based analysis with manual curation showed that 58 genes of 12,096 total genes in the* A. oryzae* genome encoded metabolic transporters. Under consensus integrative databases, 55 unambiguous metabolic transporter genes were distributed into channels and pores (7 genes), electrochemical potential-driven transporters (33 genes), and primary active transporters (15 genes). To reveal the transporter functional role, a combination of homology modeling and molecular dynamics simulation was implemented to assess the relationship between sequence to structure and structure to function. As in the energy metabolism of* A. oryzae*, the H^+^-ATPase encoded by the AO090005000842 gene was selected as a representative case study of multilevel linkage annotation. Our developed strategy can be used for enhancing metabolic network reconstruction.

## 1. Introduction

*Aspergillus oryzae* belongs to a group of filamentous fungi that has long been used for the commercial production of different industrial enzymes, such as alpha-amylases [1], proteases [2], glucoamylases [3], xylanases [4], other hydrolytic enzymes [5], and organic acids [6]. Not only does* A. oryzae* produce various biological compounds, but also it has beneficial features, such as acting as a robust host system with high production yields and acclimatization to environmental and nutritional duress [7]. In 2005, the whole genome of* A. oryzae* strain RIB40 was sequenced and annotated [8]. Very recently, the quality of the genome sequence was improved and verified using next-generation sequencing platforms, such as SOLiD [9] and Illumina MiSeq [10]. Moreover, the advancement of multilevel omics integrative analysis (genomics, transcriptomics, and proteomics) has enabled the interpretation of high-throughput data for functional annotation. In addition, the number of annotated genes in* A. oryzae* was enhanced using expressed sequence tags data [11]. Clusters of genes were then identified and annotated by oligonucleotide microarrays [12, 13] and mRNA sequencing technology [14].

Using a systems biology approach, a genome-scale metabolic network of* A. oryzae* was reconstructed based on annotated genomic data, which contains 1,314 enzyme-encoding genes including 53 metabolic transporter-associated genes [11]. Modeling of the genome-scale metabolic network of* A. oryzae* has been used to evaluate fungal biological processes and cellular physiology. However, the connectivity of metabolic networks remains incomplete because of the poor annotation of transporter genes. Among the 161 unique transport reactions, only 33% of annotated genes were identified and used in the network [11]. In metabolic pathways, transporters appear to play crucial roles in controlling the flux of molecules into and out of cells [6, 15, 16]. Additionally, several transporters regulate metabolic energy generation, delivery of essential nutrients, waste product elimination, and survival under environmental changes [17].

The techniques used for transporter annotation are often performed by sequence-based analysis using pairwise and multiple sequence alignment. Many studies of fungal transporters have relied on similarity searching between orthologous sequences using the BLASTP algorithm [18], such as investigating the gene encoding glucose transporter (hxtB–E) in the genome of* Aspergillus nidulans*. In particular, use of the ClustalW program [19] allowed for the clustering and the identification of conserved sequences and evolutionary relationship among orthologs of fungal transporters. In a study of amino acid uptake in rust fungi (plant pathogenic fungi), 60 genes were identified from rust fungal genomes and then clustered into three different transporter families, including 33 genes in yeast amino acid transporters, 20 genes in amino acid/choline transporters, and 7 genes in L-type amino acid transporters [20]. This study indicated several transporter genes in rust fungal genomes, which may play a role in interactions between plant and rust fungi [20]. However, sequence-based analysis is limited to functional annotation. For example, there is a case of two proteins, which have overall identical protein folds implying their closely related functions, but no statistically significant degree of sequence identity was observed [21]. To address such this case, structural studies through three-dimensional (3D) structure from crystallography have greatly enhanced our understanding of the potential protein function. As an example case presented in yeast, the structure of V-ATPase from* Saccharomyces cerevisiae* was determined using electron cryomicroscopy wherein the conformational changes for three functional states were observed during proton translocation [22]. Recently, the crystal structure of the phosphate transporter from* Piriformospora indica* was determined using X-ray crystallography, suggesting both proton and phosphate exit pathways and the mechanism of phosphate transport [23]. However, the number of molecules with unsolved 3D structures and unknown functions is increasing rapidly because the experimental assays to determine these properties are time-consuming and expensive. Computational approaches enable functional annotation and can be used to overcome these limitations. As observed in* A. nidulans*, the relationship between the structure and function of the subfamily of urea/H^+^ membrane transporter for the UreA gene was studied [24]. Homology models of the urea transporter were developed from the crystal structures of other organisms [25, 26] as templates combined with site-directed and classical random mutagenesis. This computational approach can be used to identify critical residues for urea transport and understand the binding, recognition, and translocation of urea [24]. However, the structure-based approaches generally rely on single static structure and do not involve dynamic information. In fact, structural dynamics can enhance functional prediction, in which the homology modeling and molecular dynamics (MD) simulation have already been extensively used as tools to further access possible functions of several specific fungal transporters (e.g., proline permease [27] and purine and pyrimidine transporters [28]). Moreover, dynamic information from MD simulation revealed the molecular mechanism of the proton pump related to conformational changes during proton translocation through H^+^-ATPase [29, 30].

As described above, current approaches can only be performed manually and specifically and cannot be used to describe the relationship between sequence, structure, and function for annotating high-throughput data of transporters. Based on experimental data of* A. oryzae*, very few reports involved in metabolic transporters, such as maltose permease [31, 32], sulphate permease [33], malic acid transporter [6], C_4_-dicarboxylate transporter [34], and uric acid-xanthine permease [35], existed. Therefore, the advanced annotation approaches can be used to increase the efficiency of transporter annotation. In this study, we developed a metabolic annotation strategy to determine the relationship between sequence, structure, and function to annotate metabolic transporters in the* A. oryzae* genome. Sequence-based analysis is used to predict transporter genes. Next, candidate transporter genes were subjected to functional classification. The transporters involved in metabolic process were manually curated by integrative analysis (i.e., integrative databases, phylogenetics, protein domains, or transporter components). In addition, the combination of homology modeling and MD simulation was used to determine the relationship between sequence to structure and structure to function. This proposed metabolic annotation strategy can be used to improve the genome-scale metabolic network of* A. oryzae* and relevant fungi.

## 2. Materials and Methods

### 2.1. Sequence Alignment Analysis for Transporter Gene Prediction

**Figure 1:**
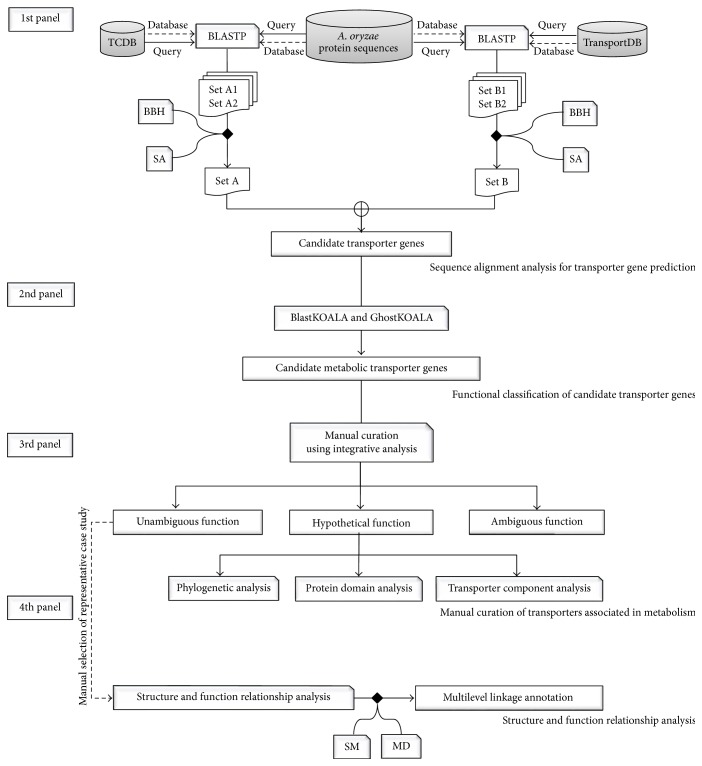
Diagram shows overall framework of a metabolic annotation strategy for linkage between sequence, structure, and function for annotating metabolic transporters in* A. oryzae* genome. In the 1st panel, Sets A and B indicate* A. oryzae* protein sequences searched against TCDB and TransportDB databases, respectively, under bidirectional best-hit analysis (BBH) and sensitivity analysis (SA). In the 4th panel, dash line implies the manual selection of a metabolic transporter from unambiguous function group as a representing case study of multilevel linkage annotation. SM and MD stand for SWISS-MODEL and molecular dynamics simulation, respectively.

To identify all possible candidate transporter genes, 12,096 protein sequences from* A. oryzae* genome [8] were searched against protein sequences from two different transporter databases that are available that is, transporter classification database (TCDB) [36] and TransportDB [37] using BLASTP (version 2.2.29^+^) [18] under bidirectional best-hit and sensitivity analysis [38] as shown in Figure 1 (1st panel). For TCDB, it is a curated transporter database of factual information from over 10,000 published references. Unique proteins in TCDB are deposited over 10,000 sequences which are classified into over 800 transporter families based on the transporter classification (TC) system according to functional and phylogenetic information [39]. In contrast, TransportDB is a relational database describing the predicted transporters based on automated annotation tool for organisms whose complete genome sequences are available [40].

### 2.2. Functional Classification of Candidate Transporter Genes

For functional classification, the candidate transporter genes obtained were submitted as dataset queries using the BlastKOALA and GhostKOALA annotation tools [41] as shown in Figure 1 (2nd panel). These are KEGG internal annotation tools for assignment of KEGG Orthology (K) number to the query protein sequences by BLAST searching against a nonredundant set of KEGG GENES, which was determined using a 50% identity cut-off [42, 43]. It is noted that GhostKOALA is suitable for annotating a large amount of metagenome sequence data by GHOSTX searching using a cut-off GHOSTX score of 100. After the submission of queries, the annotation data with assigned K numbers was downloaded and used for KEGG Mapper analysis to determine the full details of the assigned K numbers for each candidate transporter gene [41]. The function of candidate transporter gene was then manually classified into two main categories, including (i) metabolic process and (ii) nonmetabolic process. Candidate transporter genes involved in various metabolisms (i.e., energy, lipid, nucleotide, amino acid, glycan, and others) and metabolic transport processes (i.e., solute carrier family, nutrient uptake, and ion channel) were categorized into the metabolic process. Candidate transporter genes related to signaling, cellular, and genetic information were categorized into the nonmetabolic process. Candidate transporter genes with unclassified functions were categorized into the unclassified process. Only candidate transporter genes associated with the metabolic process were subsequently performed by manual curation.

### 2.3. Manual Curation of Transporters Associated with Metabolism

Candidate transporter genes categorized into the metabolic process were manually curated functions using integrative databases, including TCDB [36], KEGG [42, 43], and PFAM [44], as shown in Figure 1 (3rd panel). If transporters showed the same functions in all the three databases, they were categorized into the unambiguous function group. Otherwise, they were included in the hypothetical function group. These further required additional manual curation for transporter function. Such phylogenetic analysis combined ClustalW [45] with MEGA6 (Molecular Evolutionary Genetics Analysis, version 6.0) [46] and was manually performed to reveal evolutionary relationship of hypothetical metabolic transporter gene based on the maximum likelihood approach [47]. Alternatively, protein domain analysis was performed. Hypothetical metabolic transporter gene was manually submitted to HMMER [48] and MEME [49] and then searched for protein domains using the hidden Markov models [44, 50]. Otherwise, transporter component analysis was done. Hypothetical metabolic transporter gene was manually searched against protein sequences in TCDB based on sequence similarity to identify transporter components. Each component was afterwards curated against several protein databases (e.g., carbohydrate-active enzymes database (CAZy) [51] and Universal Protein Resource (UniProt) database [52]). Transporters showing ambiguity remained in the ambiguous function group.

### 2.4. Structure and Function Relationship Analysis

Protein structure is more evolutionarily conserved than amino acid sequence. Therefore, the analysis of 3D structures is a promising method for the functional annotation of transporters. Homology modeling was performed as shown in Figure 1 (4th panel). Initially,* A. oryzae* protein sequences belonging to the unambiguous function group were submitted as queries to the SWISS-MODEL [53] for searching the template against the Protein Data Bank (PDB) [54]. Next, a metabolic transporter from unambiguous function group that showed the highest quality with the best-identified structural template (i.e., sequence identity and percent coverage) was manually selected as the representative case study of multilevel linkage annotation. For structure-based sequence alignment of the query and template, the conserved residues between the query and template were retained in the homology model using ProMod II [55]. Remodeling was carried out by substitution of the appropriate amino acids. In order to obtain the homology protein structure, MD simulation was conducted using GROMACS version 4.5.5 [56]. Protein topology was created using the standard GROMOS96 force field parameter set 53a6 [57] and solvated based on the simple point charge water model [58]. To remove steric conflicts between atoms and to avoid high energy interactions, system energy was minimized for 2,000 steps. MD simulation was afterwards run in the NVT (constant particle number, volume, and temperature) ensemble for 100 ns with an integration time step of 1 fs. The temperature was kept constant at 298 K using the V-rescale algorithm with a time constant of 0.1 ps [59–61]. Periodic boundary conditions were applied in all directions. The real-space part of the electrostatic and Lennard-Jones interaction was set at a 1.0 nm cut-off. Long-range electrostatics were calculated using particle-mesh Ewald [62, 63] with a 0.12 nm grid and the cubic interpolation of order four in the reciprocal-space interactions. To avoid physical artifacts, the tested protocol was employed [64–66]. All bond lengths were constrained using the LINCS algorithm [67]. System visualization was performed using Visual Molecular Dynamics software [68]. The structural template was used as the reference, in which the homology model was created and simulated for comparison. At equilibrium, the trajectories were determined as the stability of global protein structure by calculating the root mean square deviation (RMSD) and root mean square fluctuation (RMSF).

## 3. Results and Discussion

Using our developed metabolic annotation strategy for transporters, we achieved four main results as described in the following. First, we describe the assessment of candidate transporter genes. Next, we present the classified functions of candidate transporter genes. Focusing on metabolic process category, we describe the manually curated transporters associated in metabolism. To this end, the structure and function relationship assessment of unambiguous metabolic transporter is discussed.

### 3.1. Assessment of Candidate Transporter Genes

Candidate transporter genes were identified by sequence alignment analysis using 12,096 protein sequences of* A. oryzae* against protein sequences in TCDB and TransportDB. We identified 129 and 23 protein sequences with one-to-one homologous relationship by bidirectional best-hit analysis in TCDB and TransportDB, respectively. These results were subsequently subjected to sensitivity analysis by varying the *E*-values as cut-offs. The *E*-values of 6*E* − 09 and 5*E* − 04 were selected as the suitable estimated cut-off values. Hereby, we obtained 112 and 18 possible transporter genes from TCDB and TransportDB, respectively. All possible transporter genes under statistical significance were overlapped and removed duplicate data. Consequently, 123 candidate transporter genes of* A. oryzae* were obtained as presented in Table 1. Full list of candidate transporter genes is provided in Table S1 in Supplementary Material available online at http://dx.doi.org/10.1155/2016/8124636.

### 3.2. Classified Functions of Candidate Transporter Genes

**Figure 2:**
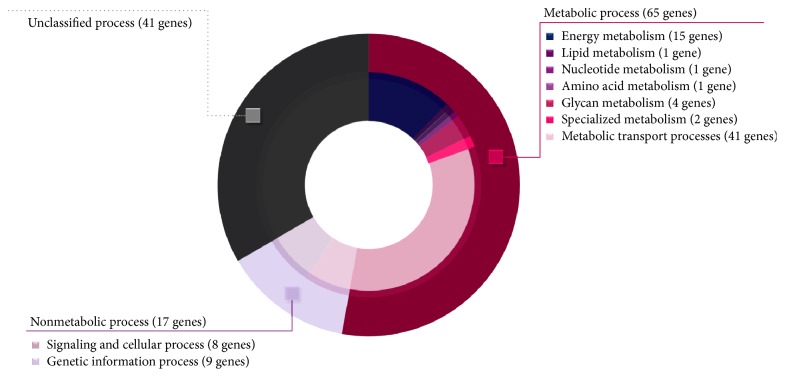
Doughnut chart illustrates different functional categories of* A. oryzae* candidate transporter genes. Outer layer shows three main functional categories (i.e., metabolic, nonmetabolic, and unclassified processes). Inner layer shows seven subcategories distributed into metabolic process and two subcategories distributed into nonmetabolic process. Ring size reflects the relative ratio of genes identified in each category.

A total of 123 candidate transporter genes were submitted as dataset queries to the BlastKOALA and GhostKOALA annotation tools. Based on the KEGG database results, 87 of the 123 submitted queries were assigned K numbers, which were manually classified into the metabolic process and nonmetabolic process categories (Table S2). As shown in Figure 2, the major category (65 of 123 candidate transporter genes) was in the metabolic process (Table S3), which was divided into seven subcategories, including 41 genes involved in metabolic transport processes, 15 genes involved in energy metabolism, 4 genes involved in glycan metabolism, and 5 genes involved in another four subcategories (Figure 2). In contrast, 17 candidate transporter genes were classified in the nonmetabolic process category, which was divided into two subcategories. These were 8 genes involved in signaling and cellular process and 9 genes involved in genetic information process. It has been reported that transporter genes involved in genetic information and cell signaling process are important in regulation level which can trigger cellular response process by transporting transcription factors, DNA binding protein, mRNA, miRNA, and other related genetic factors across compartments [69]. For candidate transporter genes with unclassified functions (41 genes), they were separated into unclassified process category.

### 3.3. Manually Curated Transporters Associated with Metabolism

Initially, 65 candidate metabolic transporter genes were manually curated to determine their functions using integrative databases, including TCDB, KEGG, and PFAM (Table S4). The results showed that the transporter functions were classified into three assigned function groups, namely, unambiguous, hypothetical, and ambiguous functions.

For the unambiguous function group, 55 of the 65 transporter genes were manually curated and found to be overlapped among the integration of three databases as summarized in Table 2. The 55 transporter genes were clustered into three classes using the TC system. Seven of the 55 transporter genes were involved in ammonium, magnesium, copper, and water transporters, which belonged to channels and pores (class 1). Most of the unambiguous function group (33 of 55 transporter genes) were involved in electrochemical potential-driven transporters (class 2), such as carbohydrate, amino acid, and nutrient uptake transporters. As example in class 2, AO090009000688 gene was curated as a nucleotide sugar transporter involved in transporting GDP-mannose, which was synthesized in the cytosol and nucleus and transported to the endoplasmic reticulum and the Golgi apparatus for mannosylation process [70]. Dean et al. demonstrated that a mutation in the gene encoding GDP-mannose transporter (*VRG4*) in* S. cerevisiae* caused a loss of mannosylation in* vrg4* mutants, leading to cell death [71]. For gene orthologs of* VRG4* identified in* Aspergillus fumigatus* [72] and* A. nidulans* [73], they were also found to be associated with polysaccharide synthesis during spore germination. In addition, three zinc transporter genes (AO090005000026, AO090011000831, and AO090026000441) corresponded to zinc tolerance and accumulation in* A. oryzae* [74]. Interestingly, large amounts of zinc could be accumulated in mycelial cells of* A. oryzae* [74]. Accordingly, this suggests that zinc transporter can be used to improve the absorption capacity of* A. oryzae* towards pollutant metals. For the other remaining manually curated genes, 15 of 55 transporter genes were functionally assigned for the primary active transporters (class 3). As seen in class 3, observably most of the transporter function utilized energy from ATP hydrolysis to transport ions through cellular membranes against a concentration gradient [29] (Table 2). For instance, AO090102001037 gene encoding proton-translocating transhydrogenase can hydrolyze ATP to transport proton through cellular membrane. Notably, this AO090102001037 gene showed evolutionary relationship among* Aspergillus* species in terms of gene sequence and expression [75].

For the hypothetical function group, 3 of the 65 transporter genes (i.e., AO090001000747, AO090023000801, and AO090005000980) were manually curated for individual transporter function by either phylogenetic, protein domain, or transporter component analysis, respectively.

**Figure 3:**
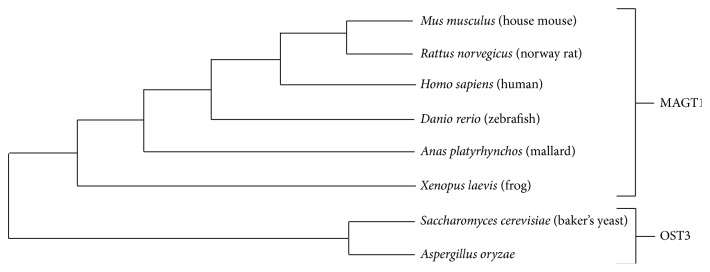
Horizontal cladogram shows an evolutionary relationship of oligosaccharyltransferase (OST3) and magnesium transporter (MAGT1) among* A. oryzae* and 7 different model organisms (i.e.,* Mus musculus*,* Rattus norvegicus*,* H. sapiens*,* Danio rerio*,* Anas platyrhynchos*,* Xenopus laevis*, and* S. cerevisiae*). The figure is generated by the MEGA6 [46] and ClustalW [45].

Performing phylogenetic analysis, the hypothetical metabolic transporter gene, for example, AO090001000747 in* A. oryzae* and oligosaccharyl transferase (OST3) in* S. cerevisiae,* showed a closer evolutionary relationship than magnesium transporter (MAGT1) in* Homo sapiens* as illustrated in Figure 3. As a result, it is promising that AO090001000747 gene is potentially encoded for the endoplasmic reticulum resident oligosaccharide transporter involved in N-glycosylation according to the function of OST3 in* S. cerevisiae* [76]. Previously, it has been reported that OST3 is a gate keeper for the secretory pathway [77] and it can catalyze the priority step in protein secretion [78]. Therefore, the significant transcriptional upregulation of AO090001000747 gene (OST3 ortholog) was accordingly reported in an* A. oryzae* alpha-amylase overproducing strain [1]. Our finding implies that AO090001000747 gene is contributed for transporting and encompassing secretory proteins, which is favorable for increasing the efficiency of commercial protein secretion in* A. oryzae*. Full details of horizontal cladogram can be seen in Figure S1.

Considering protein domain analysis, it is an alternative way for manual curation of transporter function. Once HMMER [48] and MEME [49] were used for searching the protein domains of hypothetical metabolic transporter gene, for example, AO090023000801, observably this gene contains the conserved carboxylase domain which represents a conserved region in pyruvate carboxylase and oxaloacetate decarboxylase. A report by Knuf et al. supported that AO090023000801 gene encoding pyruvate carboxylase was involved in organic acid production [6]. Besides, a manual sequence searching by TCDB [36] also supported that AO090023000801 gene encoding oxaloacetate decarboxylase was involved in sodium transport. These results thus imply that the AO090023000801 gene may have two transporter functions related to the conserved region. For the other transporter component analysis, the hypothetical metabolic transporter gene, for example, AO090005000980, was manually searched against protein sequences in TCDB [36] based on sequence similarity to identify transporter components. Accordingly, AO090005000980 gene was identified as potassium transporter (Ktr) containing three different components (i.e., the potassium-translocating protein (KtrB), regulatory protein (KtrA), and Slr1508 protein). Using CAZy [51] and UniProt [52], the protein function of Slr1508 was glycosyl transferase involved in glycosylphosphatidyl inositol anchor formation. After using PFAM [44], the results also supported that the Slr1508 protein has glycosyl transferase function. These suggest that the AO090005000980 gene may have two transporter functions relevant to the transporter components. Transporter genes showing functional ambiguity remained in the ambiguous function group (7 of 65 transporter genes), namely, genes AO090005001300, AO090120000224, AO090011000320, AO090020000415, AO090020000492, AO090010000212, and AO090012000733.

### 3.4. Structure and Function Relationship Assessment of Unambiguous Metabolic Transporter

**Figure 4:**
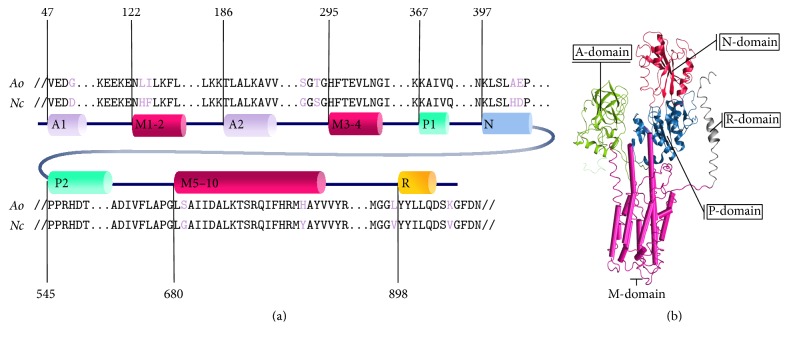
Diagram shows sequence alignment between the H^+^-ATPase in* A. oryzae* (*Ao*) and* N. crassa* (*Nc*) (PDB ID: 1MHS) [30] in (a) and structural template with five principle domains distinguished with different colors in (b). For both (a) and (b), A1-2 indicates actuator (A) domain shaded in green, P1-2 indicates the phosphorylation (P) domain shaded in blue, N indicates the nucleotide-binding (N) domain shaded in red, M1-2, M3-4, and M5–10 indicate the transmembrane (M) domain shaded in pink, and R indicates the regulatory (R) domain of the H^+^-ATPase shaded in grey.

To ensure the functional role of the unambiguous metabolic transporter, a combination of homology modeling and MD simulation was used to assess the relationship between sequence to structure and structure to function, which provides stronger evidence for functional conservation and annotation of transporter beyond sequence-based analysis. To do this, a metabolic transporter from unambiguous function group was manually selected based on the central transporter role in metabolism of* A. oryzae* with the highest sequence identity and coverage from sequence alignment analysis between the query (e.g., metabolic transporter gene) and the well-known structure and function of transporter in PDB. Among the unambiguous metabolictransporters, favorably AO090005000842 gene encoding for H^+^-ATPase was selected as a representative case study of multilevel linkage annotation due to the highest sequence identity and percent coverage between AO090005000842 gene and the well-known structure and function of the H^+^-ATPase of* Neurospora crassa*. To elaborate, AO090005000842 gene was initially submitted as a query onto the SWISS-MODEL for template searching against the PDB. According to the highest quality results among the top 10 identified templates (Table S5), the electron crystallography structure of H^+^-ATPase in* N. crassa* (PDB ID: 1MHS) [30] showed the highest sequence identity (77.47%) and percent coverage (94%). Therefore, 1MHS was used as a template for the homology modeling of* A. oryzae* H^+^-ATPase. Thus, the model was generated with detailed sequence alignment between H^+^-ATPase in* A. oryzae* and* N. crassa* (Figures 4(a) and S2). Overall, 681 residues in the five principal domains were identical in both proteins, as shown in Figure 4(b). The most homologous domain was the phosphorylation (P) domain (92.12%), followed by the cluster of 10 transmembrane helices (M1-2, M3-4, and M5–10) in the membrane domain (80.52%), the nucleotide-binding domain (72.30%), the actuator domain (64.13%), and the regulatory domain (60.53%). Additional details are shown in Table S6.

In addition to the analysis of static structures by homology modeling, MD simulation was carried out in order to evaluate structural stability during dynamics simulation and the changes in the stability of proton-transporting regions compared with template structures. The dynamics systems of both H^+^-ATPase models were created under the GROMOS96 force field and solvated in a simple point charge water model without constraints. These systems were then subjected to MD simulation for 100 ns while monitoring equilibration by examining the stability of the geometrical property (RMSD) of the H^+^-ATPase models. Subsequently, the RMSD and RMSF were calculated using the trajectories to quantify the stability and the fluctuation of the protein. The RMSD of global structures of the H^+^-ATPase in* A. oryzae* and* N. crassa* reached equilibrium after 50 ns using the quantities as shown in Figure S3. Indeed, all five principal domains in the* A. oryzae* and* N. crassa* H^+^-ATPases shared the same average RMSD over the equilibrium which indicated that the dynamic behavior of functional domains was conserved among these species (Table S7).

In fact, the proton-transport region (M-domain) of H^+^-ATPase is embedded in membrane environment. Therefore, M-domain of* A. oryzae* H^+^-ATPase embedding in palmitoyl oleoylphosphatidylcholine (POPC) lipid bilayer was conducted using the MD simulation. The insertion of M-domain into membrane was done as followed by Kandt et al. [79, 80] (Figure S4). The simulation was performed under NPT (constant particle number, pressure, and temperature) ensemble. Semi-isotropic pressure was applied by the Berendsen algorithm, at a pressure of 1 bar in both the *xy*-plane and the *z*-direction (bilayer normal) with a time constant of 3.0 ps and a compressibility of 4.5 × 10^−5^ bar^−1^ [59–61]. The simulation was run for 25 ns and the last 15 ns was used for analysis. The results showed that the average RMSD of the proton-transporting regions, M-domain embedding in POPC of* A. oryzae,* H^+^-ATPase was 0.398 ± 0.007 nm (Figure S5). This RMSD result supported that the M-domain embedding in POPC of* A. oryzae* H^+^-ATPase was consistently preserved with the corresponding regions in the initial structure of* N. crassa* H^+^-ATPase.

**Figure 5:**
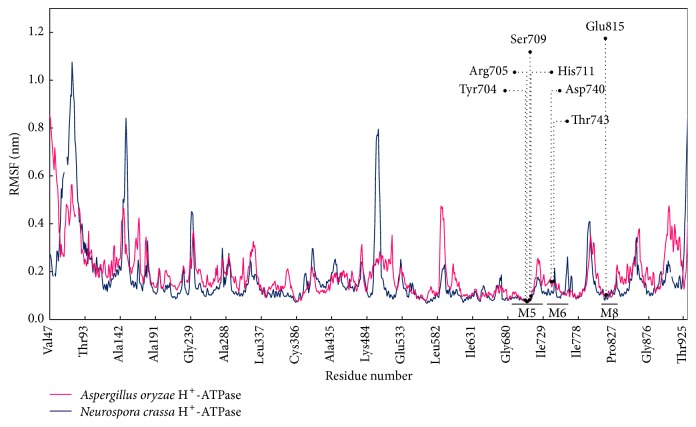
Diagram shows the comparable RMSF between the* A. oryzae* and* N. crassa* H^+^-ATPases. This graph is generated using the data in Table S8.

In addition, the proton-transporting unit of the H^+^-ATPase is defined by the presence critical proton-binding sites along proton translocation path in M-domain [81]. Such mutational H^+^-ATPase studies in plants demonstrated that substitution of Asp684 with Asn led to a defect in the conformational change for transporting protons but did not abolish the ability to bind to nucleotides and hydrolyze ATP [82]. Consistently, the substitutions of Asp730 in* N. crassa* H^+^-ATPase disrupted a salt bridge between Asp730 and Arg695, preventing the transport of protons along the proton cavity [83]. Similar structural arrangements in the proton-transporting path included positions for each conserved polar and charged residue, which may promote efficient proton transport [81]. Thus, the overall equivalent residues for proton translocation must conserve in identity and position. Therefore, fluctuations in the corresponding proton-binding sites in the* A. oryzae* H^**+**^-ATPase, including basic side chains (Arg705 and His711 on M5), acidic side chains (Asp740 on M6, Glu815 on M8), and polar side chains (Tyr704 and Ser709 on M5, Thr743 on M6), were expected to show RMSF values comparable to those of the* N. crassa* H^+^-ATPase (Figure 5). The RMSF of individual equivalent residues in the* A. oryzae* H^+^-ATPase also matched with their corresponding sites in the* N. crassa* H^+^-ATPase (Table S8). For instance, the acidic side chain Arg705 and the basic side chain Asp740 in the* A. oryzae* H^+^-ATPase fluctuated with the RMSF by approximately 0.0737 nm and 0.1530 nm, respectively, which are the corresponding sites in the* N. crassa* H^+^-ATPase, Arg695 (0.0770 nm), and Asp730 (0.1118 nm).

In accordance with the overall comparable geometrical properties, the* A. oryzae* and* N. crassa* H^+^-ATPase models were substantiated for their structural conservation at the dynamic level. Taken together, the integrative results derived from homology modeling and MD simulation supported that the proton-transporting role along the proton-transporting path in transmembrane domain was structurally conserved between H^+^-ATPases in* A. oryzae* and* N. crassa*, where functional conservation for the proton transporter is expected.

## 4. Conclusion

For the integrative multilevel annotation of metabolic transporters, we propose a metabolic annotation and assessment strategy based on sequence, structure, and function relationship as a platform for increasing the functional efficiency of transporter annotation. Of 12,096 total genes in the* A. oryzae* genome, our strategy could be used to identify 58 metabolic transporter genes. Under consensus integrative databases, 55 unambiguous metabolic transporter genes were distributed into channels and pores (7 genes), electrochemical potential-driven transporters (33 genes), and primary active transporters (15 genes). The remaining 3 hypothetical metabolic transporter genes were manually curated transporter functions by phylogenetic, protein domain, and transporter component analysis. Among the unambiguous metabolic transporter genes, the H^+^-ATPase or proton pump encoded by the AO090005000842 gene was selected as a representative case study of multilevel linkage annotation in order to reveal the transporter functional role in* A. oryzae* metabolism. Our metabolic annotation strategy can be used for improving functional annotation and enhancing cellular metabolic network and modeling in* A. oryzae* and relevant fungi.

## Supplementary Material

The detailed information of metabolic transporter genes and functions achieved from sequence- and structure-based annotation and assessment in *Aspergillus oryzae* are included in the Supplementary Material.

## Figures and Tables

**Table 1:** Number of candidate transporter genes identified by sequence alignment analysis.

Database-based annotation	*E*-value^*∗*^	Number of candidate transporter genes
TCDB	6*E* − 09	112
TransportDB	5*E* − 04	18
		123

^*∗*^Suitable estimated cut-off values.

**Table 2:** List of manually curated transporter genes and functions in unambiguous function group.

Name of transporter gene	TCID	Name of transporter function^*∗*^
Class 1: channels and pores		

AO090023000569	1.A.1.7.1	Outward-rectifier potassium channel
AO090038000314	1.A.11.3.2	Ammonium transporter
AO090003001402	1.A.35	Magnesium transporter
AO090120000141	1.A.35.5.1	Magnesium transporter
AO090120000214	1.A.56.1.4	Copper transporter
AO090011000329	1.A.8.8.8	Aquaporin
AO090023000895	1.B.8.1.1	Voltage-dependent anion channel porin

Class 2: electrochemical potential-driven transporters		

AO090003000050	2.A.1.7.1	L-fucose permease
AO090012000623	2.A.1.8.5	Nitrate transporter
AO090010000135	2.A.100.1.3	Iron-regulated transporter
AO090010000229	2.A.17.2.2	Proton-dependent oligopeptide transporter
AO090026000828	2.A.19.4.4	Sodium/potassium/calcium exchanger
AO090009000637	2.A.2.6.1	Alpha-glucoside permease
AO090003001404	2.A.20	Phosphate transporter
AO090012000901	2.A.20.2.2	Phosphate transporter
AO090103000274	2.A.22.3.2	Sodium and chloride dependent GABA transporter
AO090009000405	2.A.29.1.3	Mitochondrial adenine nucleotide translocator
AO090005000114	2.A.3.10.2	Amino acid transporter
AO090009000636	2.A.36.1.12	Sodium/hydrogen exchanger
AO090005000019	2.A.39.3.1	Allantoin permease
AO090005000455	2.A.40.5.1	Purine permease
AO090003000443	2.A.41.2.7	H^+^/nucleoside cotransporter
AO090003000920	2.A.47.2.2	Phosphate transporter
AO090026000432	2.A.49.1.3	Chloride channel
AO090005000026	2.A.5.1.1	Zinc transporter
AO090011000831	2.A.5.5.1	Zinc transporter
AO090026000441	2.A.5.7.1	Zinc transporter
AO090011000817	2.A.52.1.3	Nickel transporter
AO090003000798	2.A.53.1.2	Sodium-independent sulfate anion transporter
AO090003001119	2.A.55.1.1	High-affinity metal uptake transporter
AO090003001233	2.A.57.3.1	Nucleoside transporter
AO090005001332	2.A.59.1.1	Arsenite transporter
AO090120000217	2.A.6.6.5	Hydroxymethylglutaryl-CoA reductase
AO09M000000016	2.A.63	NADH-ubiquinone oxidoreductase
AO090001000748	2.A.66	Polysaccharide exporter
AO090010000775	2.A.7.10.2	UDP-xylose/UDP-N-acetylglucosamine transporter
AO090009000400	2.A.7.11.1	UDP-galactose transporter
AO090009000688	2.A.7.13.2	GDP-mannose transporter
AO090026000255	2.A.72.3.2	Potassium transporter
AO090005001455	2.A.97.1.4	Potassium and hydrogen ion antiporter

Class 3: primary active transporters		

AO090009000651	3.A.1.201.11	Multidrug resistance protein 1
AO090038000399	3.A.1.31.1	Possible ABC transporter permease for cobalt
AO090003000688	3.A.19.1.1	Arsenite-translocating ATPase
AO090010000482	3.A.2	V-type ATPases
AO09M000000001	3.A.2.1.3	F-type ATPase
AO090012000797	3.A.2.2.3	V-type ATPase
AO090038000088	3.A.3.1.7	P-type ATPase
AO090012000773	3.A.3.10.1	P-type ATPase
AO090038000322	3.A.3.2.2	P-type ATPase
AO090005000842	3.A.3.3.6	Plasma membrane proton ATPase
AO09M000000013	3.D.1.2.1	NADH dehydrogenase
AO09M000000015	3.D.1.6.2	NADH-ubiquinone oxidoreductase
AO090102001037	3.D.2.4.1	Proton-translocating transhydrogenase
AO090010000475	3.D.3.2.1	Cytochrome b-c1 complex subunit Rieske
AO09M000000014	3.D.4.8.1	Cytochrome oxidase

^*∗*^Names of transporter functions are based on KEGG, PFAM, and UniProt databases.
