# Supplementary material for: Sequence- and Structure-Based Functional Annotation and Assessment of Metabolic Transporters in Aspergillus oryzae: A Representative Case Study
Source: Biomed Res Int. 2016 May 4;2016:8124636. doi: 10.1155/2016/8124636 (PMC4870676; doi:10.1155/2016/8124636)
Supplement: Supplementary file 1 — The detailed information of metabolic transporter genes and functions achieved from sequence- and structure-based annotation and assessment in Aspergillus oryzae are included in the Supplementary Material. [file 8124636.f1.pdf]

Supporting Information

Sequence- and Structure-Based Functional Annotation and Assessment of Metabolic Transporters in *Aspergillus oryzae*: A Representative Case Study

Nachon Raethong[1], Jirasak Wong-ekkabut[2,3], Kobkul Laoteng[4], and Wanwipa Vongsangnak[1,3*]

[1] *Department of Zoology, Faculty of Science, Kasetsart University, Bangkok, Thailand*
[2] *Department of Physics, Faculty of Science, Kasetsart University, Bangkok, Thailand*
[3] *Computational Biomodelling Laboratory for Agricultural Science and Technology (CBLAST), Faculty of Science, Kasetsart University, Bangkok, Thailand*
[4] *Food Biotechnology Research Unit, National Center for Genetic Engineering and Biotechnology (BIOTEC), National Science and Technology Development Agency (NSTDA), Pathum Thani, Thailand*

[*] *Correspondence should be addressed to Wanwipa Vongsangnak; wanwipa.v@ku.ac.th*

Contents

Figure S1. Horizontal cladogram of evolutionary relationship among MAGT1, OST3, and AO090001000747 gene 2

Figure S2. Overall structure-based sequence alignment between the *A. oryzae* and *N. crassa* H^+-ATPases 4

Figure S3. Comparable RMSD variations of global structures between *A. oryzae* and *N. crassa* H^+-ATPases on simulation time 5

Figure S4. Initial snapshot of the MD simulation of M-domain of *A. oryzae* H^+-ATPase integrated in palmitoyl oleoylphos phatidylcholine (POPC) lipid bilayer 6

Figure S5. The RMSD of M-domain of *A. oryzae* H^+-ATPase integrated in palmitoyl oleoylphos phatidylcholine (POPC) lipid bilayer 7

Table S1. List of 123 candidate transporter genes with predicted functions from TCDB and TransportDB 8

Table S2. List of 123 candidate transporter genes with assigned K numbers and KEGG orthology definition 15

Table S3. List of 65 transporter genes and functions involved in *A. oryzae* metabolism 19

Table S4. List of 65 transporter genes with curated functions from integrative databases of TCDB, KEGG, and PFAM 21

Table S5. List of top 10 identified templates for *A. oryzae* H^+-ATPase encoded by AO090005000842 gene 23

Table S6. List of conserved catalytic domains between the *A. oryzae* and *N. crassa* H^+-ATPases with percentage of identity 24

Table S7. List of average RMSD over the equilibrium of five principal regions in *A. oryzae* and *N. crassa* H^+-ATPases 25

Table S8. Comparable RMSF between *A. oryzae* and *N. crassa* H^+-ATPases 26

Figure S1. Horizontal cladogram of evolutionary relationship among MAGT1, OST3, and AO090001000747 gene

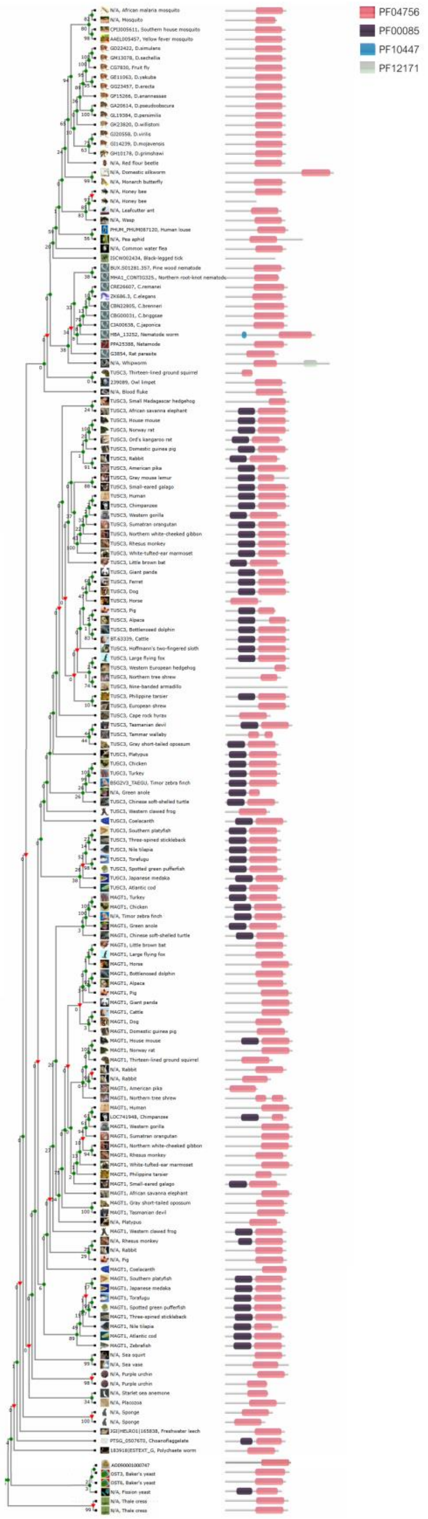
PF04756
PF00085
PF10447
PF12171

Figure S2. Overall structure-based sequence alignment between the *A. oryzae* and *N. crassa* H^{+}-ATPases

```
                                                         A-domain (1)
A. oryzae   MPNIQHSLDTTGAPHGDGASSSQDYVDPKEQFVTRASAEYKSFTENVEDGEDVENIDTLIEELESLDGNLDYNDSGQDEKSTPC---CISEELLQTDPST  97
N. crassa   ----------MADHSASGAPALSTNIESGKFDEKAAEAAAYQPKPKVEDDED-EDIDALIEDLESHDG---HDAEEEEEEATPGGGRVVPEDMLQTDTRV  86

                                 M-domain (1)                                                       A-domain (2)
A. oryzae   GLTEPEVLLRRKKYGLNQMKEEKENLILKFLSYFVGPVQFVMEAAAILAAGLQDWVDFGVICALLILNASVGFIQEFQAGSIVDELKKTLALKAVVLRNS  197
N. crassa   GLTSEEVVQRRRKYGLNQMKEEKENHFLKFLGFFVGPIQFVMEGAAVLAAGLEDWVDFGVICGLLLLNAVVGFVQEFQAGSIVDELKKTLALKAVVLRDG  186

A. oryzae   HLAEVDASDVVPGDVLEIEEGTIIPADGRILSSSV-LQVDQSGITGESLAVDKADGDTCYSSSAVKHGHARLVVTATGDYTFVGRAAALVSAATSGTGHF  296
N. crassa   TLKEIEAPEVVPGDILQVEEGTIIPADGRIVTDDAFLQVDQSALTGESLAVDKHKGDQVFASSAVKRGEAFVVITATGDNTFVGRAAALVNAASGGSGHF  286

            M-domain (2)                                                                        P-domain (1)
A. oryzae   TEVLNGISIVLLVLVIMTLLVVWVSSFYRSNGIVTILEFTLAITMIGVPVGLPAVVTTTMAVGAAYLAKKKAIVQRLSAIESLAGVEILCSDKTGTLTRN  396
N. crassa   TEVLNGIGTILLILVIFTLLIVWVSSFYRSNPIVQILEFTLAITIIGVPVGLPAVVTTTMAVGAAYLAKKKAIVQKLSAIESLAGVEILCSDKTGTLTKN  386

            N-domain
A. oryzae   KLSLAEPYTVPGVTSEELMLTACLAASRKKKGMDPIDRAFLRALKGYPEAKKALTQYKKLEFFPFDPVSKKVTAVVQSPHGERIVCMKGAPIFVLNTVKK  496
N. crassa   KLSLHDPYTVAGVDPEDLMLTACLAASRKKKGIDAIDKAFLKSLKYYPRAKSVLSKYKVLQFHPFDPVSKKVVAVVESPQGERITCVKGAPLFVLKTVEE  486

                                                          P-domain (2)
A. oryzae   DHPISEGVETAYMSKVADFAVRGFRSLGVARKCSEGEWEILGIMPCSDPPRHDTAKTIHEAKSLGLSIKMLTGDAVGIARETSRQLGLGTNVYNAERLGL  596
N. crassa   DHPIPEEVDQAYKNKVAEFATRGFRSLGVARKRGEGSWEILGIMPCMDPPRHDTYKTVCEAKTLGLSIKMLTGDAVGIARETSRQLGLGTNIYNAERLGL  586

                                                                                                 M-domain (3)
A. oryzae   GGKGTMPGSQVYDFVEAADGFAEVFPQHKYNVVDILQQRGYLVAMTGDGVNDAPSLKKADAGIAVEGSSDAARTAADIVFLAPGLSAIIDALKTSRQIFH  696
N. crassa   GGGGDMPGSEVYDFVEAADGFAEVFPQHKYNVVEILQQRGYLVAMTGDGVNDAPSLKKADTGIAVEGSSDAARSAADIVFLAPGLGAIIDALKTSRQIFH  686

A. oryzae   RMHAYVVVYRIALSLHLEIFLGLWIAIMNESLNIQLVVFIAIFADIATLAIAYDKAPYSRTPVKWNLPRLWGMSVLLGIVLAIGTWVTLSTMLSGGEQGGI  796
N. crassa   RMYAYVVVYRIALSIHLEIFLGLWIAILNRSLNIELVVFIAIFADVATLAIAYDNAPYSQTPVKWNLPKLWGMSVLLGVVLAVGTWITVTTMYAQGENGGI  786

                                                                                                      R-domain
A. oryzae   VQNFGKRDEVLFLEISLTENWLIFITRAEGPLWSSVPSWQLTGAILVVDLMATFFCLFGWFVGGQTSIVTVVRTWVFSIGVFCVMGGLYYLLQDSKGFDN  896
N. crassa   VQNFGNMDEVLFLQISLTENWLIFITRANGPFWSSIPSWQLSGAIFLVDILATCFTIWGWFEHSDTSIVAVVRIWIFSFGIFCIMGGVYYILQDSVGFDN  886

A. oryzae   IMNGRWPGSKASRQRQKEDFVVSMQRTSTLHEKSSLNGTDTGTELPR                                                       943
N. crassa   LMHGKSPKGN-QKQRSLEDFVVSLQRVSTQHEKSQ------------                                                       920
```

Figure S3. Comparable RMSD variations of global structures between *A. oryzae* and *N. crassa* H^+-ATPases on simulation time

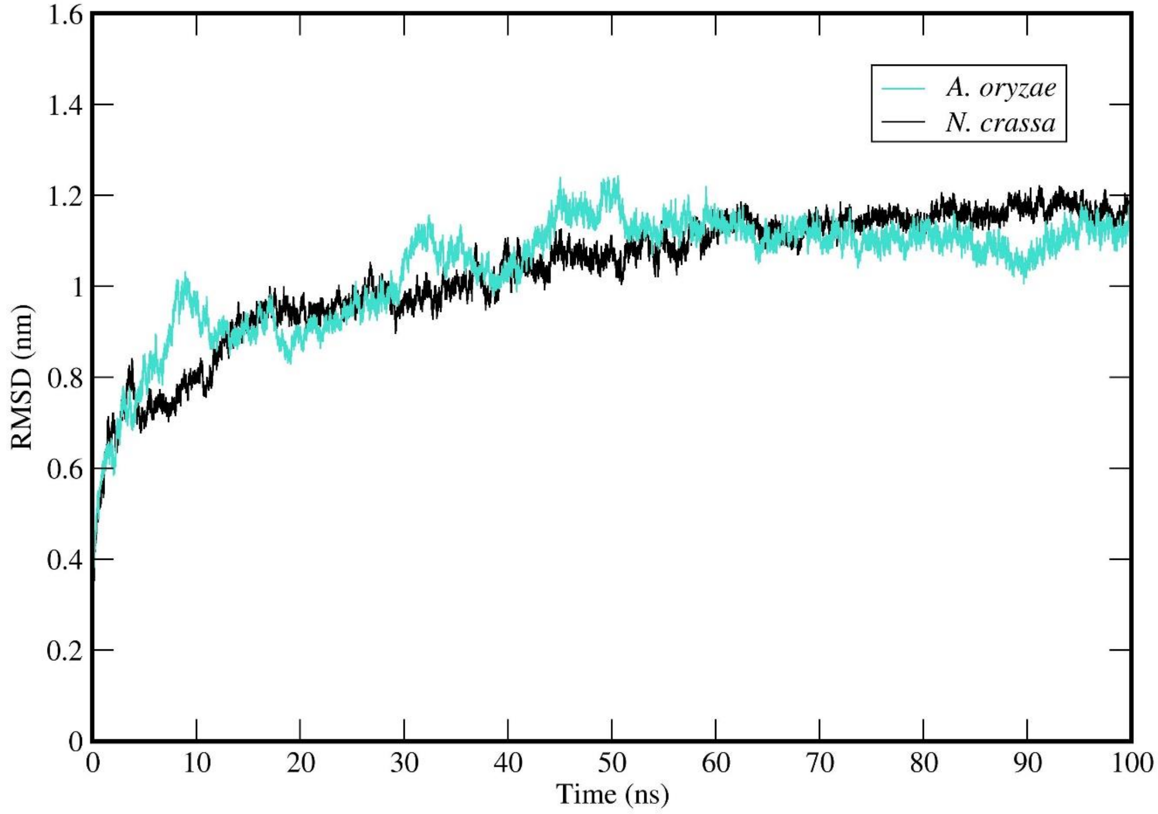

Figure S4. Initial snapshot of the MD simulation of M-domain of *A. oryzae* H^+-ATPase integrated in palmitoyl oleoylphos phatidylcholine (POPC) lipid bilayer

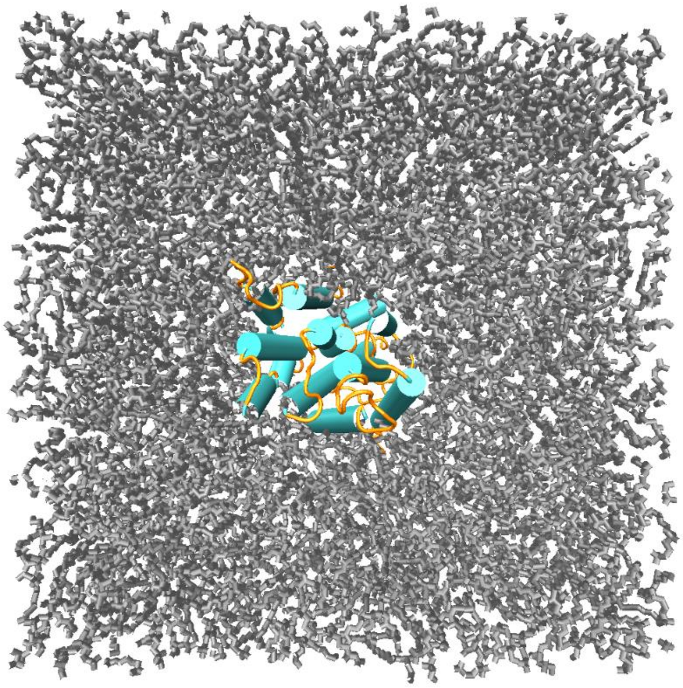

Note: The simulation system consisted of 349 residues of the protein, 477 POPC lipids, 55,570 water molecules and 2 potassium ions. The total number of atoms was 194,970 in the system with box dimensions of 12.86 x 12.86 x 14.98 nm^3. The temperature of the system was maintained at 298 K. Protein helices are shown in cyan and loops in orange. POPC is drawn as grey spheres. Water and ions are omitted for clarity.

Figure S5. The RMSD of M-domain of *A. oryzae* H^+-ATPase integrated in palmitoyl oleoylphos phatidylcholine (POPC) lipid bilayer

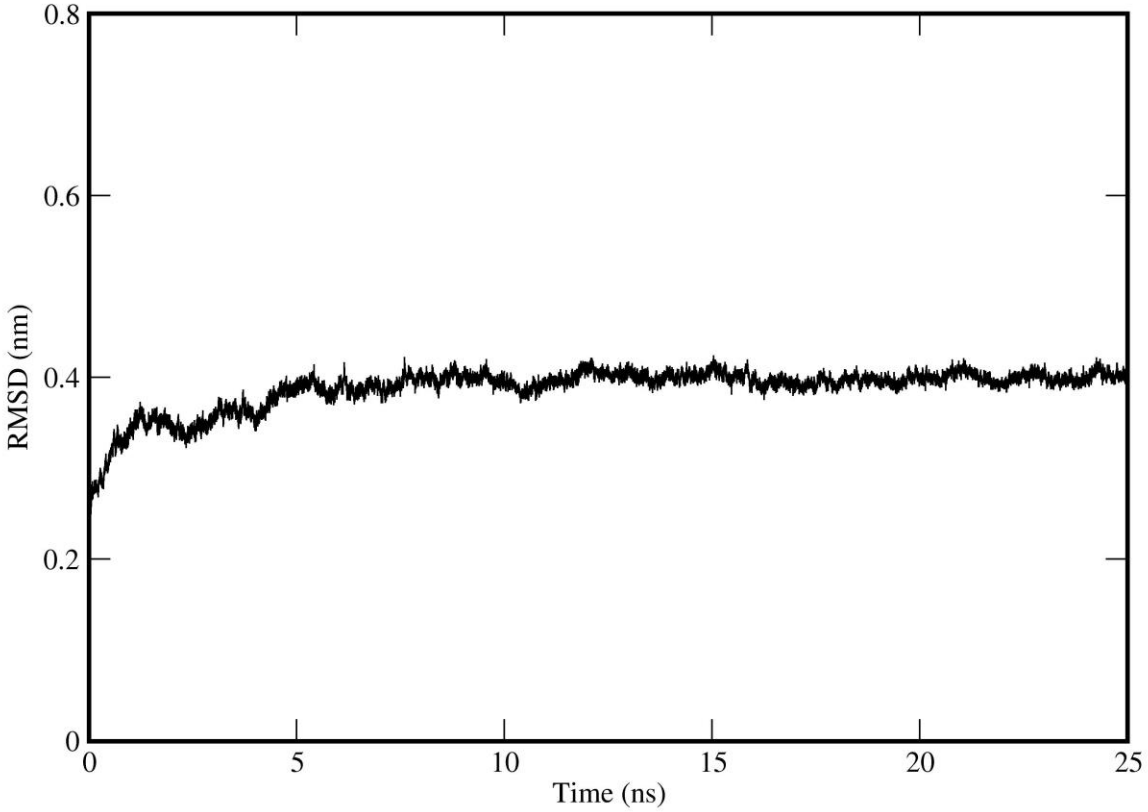

Table S1. List of 123 candidate transporter genes with predicted functions from TCDB and TransportDB

Gene name*	Database	TCID	Predicted function**	E-value	%Alignment
AO090001000091	TCDB	9.C.6	Mitochondrial organic anion channel family	1.00E-24	84.32%
	TCDB	9.C.6	Mitochondrial organic anion channel family	8.00E-25	87.57%
AO090001000747	TCDB	9.A.45.1.1	Magnesium transporter, MagT1	2.00E-23	97.26%
	TCDB	9.A.45.1.2	Tumor suppressor candidate 3 isoform a, Tusc3a	6.00E-24	76.44%
AO090003000050	TCDB	2.A.1.7.1	L-fucose permease	1.00E-54	87.37%
	TCDB	2.A.1.7.1	L-fucose permease	6.00E-55	96.80%
AO090003000307	TCDB	2.A.50.1.1	Glycerol: proton symporter	5.00E-167	94.32%
	TCDB	2.A.50.1.1	Glycerol: proton symporter	6.00E-171	96.43%
AO090003000443	TCDB	2.A.41.2.7	Proton-nucleoside cotransporter CNT1/CNT2	9.00E-177	98.36%
	TCDB	2.A.41.2.7	Proton-nucleoside cotransporter CNT1/CNT2	5.00E-177	98.85%
AO090003000454	TCDB	9.A.50.1.1	tRNA nuclear export machinery	8.00E-140	100.00%
	TCDB	9.A.50.1.1	tRNA nuclear export machinery	2.00E-135	100.00%
AO090003000460	TCDB	5.B.1.1.5	NADPH oxidase 5 ,Nox5	2.00E-57	98.00%
	TCDB	5.B.1.1.1	Phagocyte NADPH oxidase family	1.00E-92	89.47%
AO090003000506	TCDB	3.A.5.8.1	General secretory pathway (Sec-SRP) complex	2.00E-21	96.67%
	TCDB	3.A.5.8.1	General secretory pathway (Sec-SRP) complex	9.00E-32	100.00%
AO090003000688	TCDB	3.A.19.1.1	Arsenite-translocating ATPase	3.00E-117	96.46%
	TCDB	3.A.19.1.1	Arsenite-translocating ATPase	7.00E-119	92.53%
	TransportDB	3.A.4	Arsenical pump-driving ATPase	2.00E-59	93.21%
	TransportDB	3.A.4	Arsenical pump-driving ATPase	1.00E-25	97.64%
AO090003000693	TCDB	8.A.32.1.1	Beta-secretase 1	6.00E-16	88.16%
	TCDB	8.A.32.1.1	Beta-secretase 1	3.00E-16	76.45%
AO090003000798	TCDB	2.A.53.1.2	Sulfate permease	0	95.49%
	TCDB	2.A.53.1.2	Sulfate permease	0	97.68%
AO090003000854	TCDB	2.A.21.6.1	Urea active transporter	0	96.97%
	TCDB	2.A.21.6.1	Urea active transporter	0	95.78%
AO090003000920	TCDB	2.A.47.2.2	Vacuolar low affinity phosphate transporter, Pho91	0	100.00%
	TCDB	2.A.47.2.2	Vacuolar low affinity phosphate transporter, Pho91	0	100.00%
AO090003001119	TCDB	2.A.55.1.1	High-affinity metal uptake transporter, Smf1p	3.00E-163	91.84%
	TCDB	2.A.55.1.1	High-affinity metal uptake transporter, Smf1p	5.00E-168	97.57%
	TransportDB	2.A.55	Manganese transport protein, MntH	7.00E-90	84.46%
	TransportDB	2.A.55	Manganese transport protein, MntH	6.00E-41	92.88%
AO090003001232	TCDB	3.A.1.212.2	ABC mitochondrial peptide/MDR half transporter	0	76.43%
	TCDB	3.A.1.212.1	Mitochondrial peptide exporter, Mdl1p	0	85.90%
AO090003001233	TCDB	2.A.57.3.1	Nucleoside transporter, Fun26p	2.00E-45	96.19%
	TCDB	2.A.57.3.1	Nucleoside transporter, Fun26p	3.00E-44	89.17%
AO090003001402	TransportDB	1.A.35	Magnesium transport permease	1.00E-24	90.26%
	TransportDB	1.A.35	Magnesium transport permease	1.00E-24	90.71%
AO090003001404	TransportDB	2.A.20	Phosphate symporter	6.00E-65	96.60%
	TransportDB	2.A.20	Phosphate symporter	6.00E-66	96.49%

Gene name*	Database	TCID	Predicted function**	E-value	%Alignment
AO090003001489	TCDB	5.A.3.3.3	Anaerobic dimethylsulfoxide reductase	5.00E-13	89.99%
	TCDB	5.A.3.6.1	Arsenite oxidase, AoxAB	1.00E-24	79.88%
AO090005000019	TCDB	2.A.39.3.6	Benzyl-hydantoin:cation symporter-1, Mhp1	8.00E-23	93.90%
	TCDB	2.A.39.3.1	Allantoin permease	3.00E-154	79.69%
AO090005000026	TCDB	2.A.5.1.1	High affinity zinc uptake transporter, Zrt1	2.00E-139	99.72%
	TCDB	2.A.5.1.1	High affinity zinc uptake transporter, Zrt1	8.00E-140	97.34%
AO090005000114	TCDB	2.A.3.10.7	Amino acid (Asn/Gln) permease	7.00E-162	99.83%
	TCDB	2.A.3.10.2	General amino acid permease	0	90.70%
AO090005000455	TCDB	2.A.40.4.1	High affinity uric acid-xanthine permease, UapA	0	96.42%
	TCDB	2.A.40.5.1	Putative purine permease, YbbY	0	93.97%
	TransportDB	2.A.40	Nucleobase/ascorbate transporter	1.00E-68	97.65%
	TransportDB	2.A.40	Nucleobase/ascorbate transporter	3.00E-19	93.86%
AO090005000714	TCDB	2.A.21.9.2	Sodium symporter	3.00E-21	18.71%
	TCDB	2.A.21.9.2	Sodium symporter	3.00E-21	35.99%
AO090005000775	TCDB	2.A.43.1.1	Lysosomal cystine transporter	4.00E-30	81.78%
	TCDB	2.A.43.1.1	Lysosomal cystine transporter	2.00E-32	48.50%
AO090005000842	TCDB	3.A.3.3.6	Plasma menbrane ATPase (proton transporter)	0	97.88%
	TCDB	3.A.3.3.1	Plasma menbrane ATPase (proton transporter)	0	94.78%
AO090005000980	TCDB	2.A.38.4.5	Sodium dependent potassium uptake transporter	2.00E-12	59.07%
	TCDB	2.A.38.4.5	Sodium dependent potassium uptake transporter	1.00E-12	74.29%
AO090005001175	TCDB	3.A.20.1.1	Peroxisomal targeting signal 2 receptor	2.00E-55	97.46%
	TCDB	3.A.20.1.2	Peroxin complex	3.00E-75	94.95%
AO090005001209	TCDB	9.A.34.1.1	This family is currently empty	5.00E-144	93.53%
	TCDB	9.A.34.2.1	This family is currently empty	6.00E-140	88.62%
AO090005001300	TCDB	2.A.7.22.2	4-amino-4-deoxy-L-arabinose-phosphoundecaprenol flippase	1.00E-135	99.62%
	TCDB	2.A.7.22.2	4-amino-4-deoxy-L-arabinose-phosphoundecaprenol flippase	5.00E-136	99.61%
AO090005001332	TCDB	2.A.59.1.1	Plasma membrane arsenite exporter	6.00E-86	88.86%
	TCDB	2.A.59.1.2	Arsenite/antimonite exporter	2.00E-96	95.09%
	TransportDB	2.A.59	Arsenical resistance-3 family	2.00E-108	94.17%
	TransportDB	2.A.59	Arsenical resistance-3 family	1.00E-44	92.23%
AO090005001365	TCDB	2.A.1.2.35	Galactose-proton symporter, GalP	5.00E-139	99.28%
	TCDB	2.A.1.2.35	Galactose-proton symporter, GalP	6.00E-156	99.81%
AO090005001455	TCDB	2.A.97.1.4	Potassium and hydrogen ion antiporters	2.00E-108	77.72%
	TCDB	2.A.97.1.4	Potassium and hydrogen ion antiporters	1.00E-105	84.14%
AO090005001573	TCDB	3.A.1.204.9	Putative multidrug/pigment exporter, Adp1	0	99.09%
	TCDB	3.A.1.204.9	Putative multidrug/pigment exporter, Adp1	0	98.95%
AO090005001624	TCDB	9.A.14.1.1	Red-sensitive opsin	3.00E-126	98.35%
	TCDB	9.A.14.1.1	Red-sensitive opsin	2.00E-126	99.18%
AO090009000002	TCDB	2.A.16.2.2	Malate transporter	1.00E-31	23.37%
	TCDB	2.A.16.2.2	Malate transporter	5.00E-32	18.24%
AO090009000183	TCDB	9.A.2.1.1	Endosomal membrane protein 70, TMN2p	0	62.04%
	TCDB	9.A.2.1.1	Endosomal membrane protein 70, TMN2p	0	64.69%

Gene name*	Database	TCID	Predicted function**	E-value	%Alignment
AO090009000197	TCDB	9.B.1.1.1	CAAX prenyl protease 1 homolog	1.00E-39	53.78%
	TCDB	9.B.1.1.1	CAAX prenyl protease 1 homolog	9.00E-40	62.93%
AO090009000400	TCDB	2.A.7.11.1	UDP-galactose:UMP antiporter	2.00E-37	93.30%
	TCDB	2.A.7.11.4	Golgi UDP-galactose/UDP-glucose:UDP antiporter	6.00E-39	95.78%
AO090009000405	TCDB	2.A.29.1.3	ADP/ATP translocase 1	1.00E-164	99.68%
	TCDB	2.A.29.1.7	ADP:ATP carrier 2, Aac2	2.00E-175	93.08%
AO090009000582	TCDB	4.C.1.1.3	Putative long chain fatty acid transporter	2.00E-98	86.23%
	TCDB	4.C.1.1.5	Peroxysomal fatty acyl CoA synthase	8.00E-102	85.00%
AO090009000622	TCDB	8.A.34.1.1	Endophilin A1	1.00E-15	52.11%
	TCDB	8.A.34.1.1	Endophilin A1	2.00E-15	69.60%
AO090009000636	TCDB	2.A.36.1.12	Endosomal/prevacuolar sodium/hydrogen exchanger	0	94.75%
	TCDB	2.A.36.1.12	Endosomal/prevacuolar sodium/hydrogen exchanger	0	89.26%
AO090009000637	TCDB	2.A.2.6.1	General alpha-glucoside permease	3.00E-116	96.39%
	TCDB	2.A.2.6.1	General alpha-glucoside permease	2.00E-107	97.65%
AO090009000651	TCDB	3.A.1.201.11	Multidrug resistance protein 1	0	100.00%
	TCDB	3.A.1.201.11	Multidrug resistance protein 1	0	98.59%
AO090009000668	TCDB	8.A.34.2.1	Phosphate transporter	2.00E-105	99.23%
	TCDB	8.A.34.2.1	Phosphate transporter	1.00E-105	98.49%
AO090009000677	TCDB	8.A.13.1.1	Tetratricopeptide repeat protein 1	2.00E-139	91.75%
	TCDB	8.A.13.1.1	Tetratricopeptide repeat protein 1	6.00E-145	87.10%
AO090009000688	TCDB	2.A.7.13.3	Golgi GDP-mannose:GDP antiporter, GONST1	2.00E-49	91.85%
	TCDB	2.A.7.13.2	Golgi GDP-mannose transporter, VRG4	2.00E-138	82.75%
AO090010000135	TCDB	2.A.100.1.3	Ferroportin1 or Iron-regulated protein1, 9FPN1	9.00E-40	86.88%
	TCDB	2.A.100.1.3	Ferroportin1 or Iron-regulated protein1, 9FPN1	2.00E-42	91.22%
AO090010000212	TCDB	9.B.102.4.1	Rhodanese domain protein	2.00E-12	75.87%
	TCDB	9.B.102.4.1	Rhodanese domain protein	5.00E-13	39.57%
AO090010000229	TCDB	2.A.17.2.1	Probable peptide transporter	6.00E-175	95.71%
	TCDB	2.A.17.2.2	Peptide transporter	0	91.18%
AO090010000475	TCDB	3.D.3.2.1	Cytochrome b-c1 complex subunit Rieske	1.00E-78	82.70%
	TCDB	1.A.24.1.1	Cytochrome b-c1 complex subunit Rieske	4.00E-90	85.12%
AO090010000482	TransportDB	3.A.2	V-type ATPases	0	99.58%
	TransportDB	3.A.2	V-type ATPases	0	96.91%
AO090010000592	TCDB	8.A.5.1.3	Voltage-gated potassium channel subunit beta-1	2.00E-106	96.29%
	TCDB	8.A.5.1.3	Voltage-gated potassium channel subunit beta-1	8.00E-107	81.55%
AO090010000775	TCDB	2.A.7.10.2	UDP N-acetylglucosamine antiporter	5.00E-41	89.22%
	TCDB	2.A.7.10.1	UDP N-acetylglucosamine transporter	1.00E-52	97.56%
AO090011000202	TCDB	3.D.5.1.1	Sodium translocating NADH-quinone reductase	1.00E-12	58.89%
	TCDB	3.D.5.1.1	Sodium translocating NADH-quinone reductase	1.00E-11	64.13%
AO090011000320	TCDB	9.A.6.1.1	ATP release protein	0	95.01%
	TCDB	9.A.6.1.1	ATP release protein	0	98.91%
AO090011000329	TCDB	1.A.8.8.8	Aquaporin	9.00E-26	90.49%
	TCDB	1.A.8.6.2	Aquaporin	3.00E-56	80.62%

Gene name*	Database	TCID	Predicted function**	E-value	%Alignment
AO090011000535	TCDB	9.B.82.1.5	Putative heavy metal transporter	8.00E-25	94.68%
	TCDB	9.B.82.1.2	Putative heavy metal transporter	9.00E-72	82.65%
AO090011000587	TCDB	2.A.1.2.11	Amine transporter	7.00E-10	67.73%
	TCDB	2.A.1.2.29	Synaptic vesicular amine transporter	6.00E-11	51.36%
AO090011000638	TCDB	1.A.33.1.2	Heat shock protein-70 homologue	0	97.41%
	TCDB	1.A.33.1.2	Heat shock protein-70 homologue	0	80.09%
AO090011000649	TCDB	2.A.39.2.4	Cytosine-purine permease	0	100.00%
	TCDB	2.A.39.2.4	Cytosine-purine permease	0	100.00%
AO090011000817	TCDB	2.A.52.1.3	Nickel transporter	2.00E-60	55.51%
	TCDB	2.A.52.1.3	Nickel transporter	1.00E-73	85.43%
	TransportDB	2.A.52	Nickel permease	2.00E-58	81.07%
	TransportDB	2.A.52	Nickel permease	4.00E-44	56.99%
AO090011000912	TCDB	2.A.7.25.7	Magnesium transmembrane transporter	0	99.85%
	TCDB	2.A.7.25.7	Magnesium transmembrane transporter	0	98.99%
AO090012000168	TCDB	1.A.17.2.1	Anoctamin-like protein	6.00E-37	73.39%
	TCDB	1.A.17.2.1	Anoctamin-like protein	2.00E-37	52.70%
AO090012000315	TCDB	2.A.67.1.2	Tetra peptide transporter	0	99.22%
	TCDB	2.A.67.1.2	Tetra peptide transporter	0	97.45%
AO090012000623	TCDB	2.A.1.8.5	Nitrate transporter	0	99.80%
	TCDB	2.A.1.8.5	Nitrate transporter	0	99.61%
AO090012000773	TCDB	3.A.3.10.1	P-type ATPase	0	97.61%
	TCDB	3.A.3.10.1	P-type ATPase	0	99.09%
AO090012000787	TCDB	8.A.21.1.1	Erythrocyte band 7 integral membrane protein	4.00E-36	70.93%
	TCDB	8.A.21.1.1	Erythrocyte band 7 integral membrane protein	3.00E-33	84.03%
AO090012000797	TCDB	3.A.2.2.3	V-type ATPase	2.00E-129	100.00%
	TCDB	3.A.2.2.3	V-type ATPase	1.00E-129	100.00%
AO090012000865	TCDB	8.A.21.2.1	Membrane-bound protease	4.00E-33	41.97%
	TCDB	8.A.21.2.1	Membrane-bound protease	1.00E-27	68.42%
AO090012000901	TCDB	2.A.20.2.2	Phosphate transporter	8.00E-176	99.65%
	TCDB	2.A.20.2.1	Phosphate transporter	0	99.49%
AO090012000997	TCDB	1.A.9.5.2	Gamma-aminobutyric acid receptor subunit beta-2	7.00E-48	98.31%
	TCDB	1.A.9.5.2	Gamma-aminobutyric acid receptor subunit beta-2	3.00E-48	99.15%
AO090012001026	TCDB	8.A.27.1.4	Phospholipid transporter	1.00E-68	90.37%
	TCDB	8.A.27.1.2	Cell division control protein 50	9.00E-114	89.51%
AO090020000192	TCDB	2.A.1.25.1	Acetyl-coenzyme A transporter	6.00E-105	89.60%
	TCDB	2.A.1.25.1	Acetyl-coenzyme A transporter	7.00E-100	84.70%
AO090020000415	TCDB	5.A.4.1.1	This family is currently empty	4.00E-75	82.07%
	TCDB	5.A.4.1.1	This family is currently empty	6.00E-80	92.67%
AO090020000492	TCDB	3.B.1.1.5	Methylmalonyl-CoA decarboxylase	9.00E-71	84.83%
	TCDB	3.B.1.1.2	Methylmalonyl-CoA decarboxylase	4.00E-77	96.27%
AO090020000572	TCDB	3.A.1.211.5	Surfactant-secreting porter, ABCA3	8.00E-120	68.72%
	TCDB	3.A.1.211.5	Surfactant-secreting porter, ABCA3	2.00E-118	67.84%

Gene name*	Database	TCID	Predicted function**	E-value	%Alignment
AO090023000569	TCDB	1.A.1.7.1	Outward-rectifier potassium channel	2.00E-55	69.09%
	TCDB	1.A.1.7.1	Outward-rectifier potassium channel	1.00E-55	62.08%
AO090023000590	TCDB	3.A.3.9.6	P-type ATPase	0	98.39%
	TCDB	3.A.3.9.5	P-type ATPase	0	92.82%
AO090023000801	TCDB	3.B.1.1.1	Oxaloacetate decarboxylase	1.00E-86	51.13%
	TCDB	3.B.1.1.1	Oxaloacetate decarboxylase	2.00E-84	99.15%
AO090023000892	TCDB	9.B.17.1.1	Vesicle-associated membrane protein	8.00E-15	34.17%
	TCDB	9.B.17.1.1	Vesicle-associated membrane protein	5.00E-15	37.45%
AO090023000895	TCDB	1.B.8.1.1	Voltage-dependent anion channel porin	3.00E-59	99.42%
	TCDB	1.B.8.1.1	Voltage-dependent anion channel porin	2.00E-59	99.65%
AO090026000231	TCDB	9.B.45.1.3	Protein kinase	1.00E-14	60.10%
	TCDB	9.B.45.1.3	Protein kinase	1.00E-14	50.20%
AO090026000255	TCDB	2.A.72.3.2	Potassium transporter	0	89.74%
	TCDB	2.A.72.3.2	Potassium transporter	0	90.26%
	TransportDB	2.A.72	Potassium transporter	1.00E-96	85.54%
	TransportDB	2.A.72	Potassium transporter	2.00E-88	83.73%
AO090026000404	TCDB	4.C.2.1.1	Carnitine O-acetyltransferase	2.00E-108	96.23%
	TCDB	4.C.2.1.1	Carnitine O-acetyltransferase	3.00E-114	96.96%
AO090026000432	TCDB	2.A.49.1.3	Chloride channel protein	5.00E-169	87.17%
	TCDB	2.A.49.2.3	Chloride channel protein	0	90.71%
AO090026000441	TCDB	2.A.5.7.1	Zinc transporter	3.00E-51	100.00%
	TCDB	2.A.5.7.1	Zinc transporter	2.00E-51	100.00%
AO090026000611	TCDB	3.A.7.7.1	DNA topoisomerase I	2.00E-17	86.96%
	TCDB	3.A.7.7.1	DNA topoisomerase I	6.00E-14	78.43%
AO090026000661	TCDB	3.A.1.210.2	Metal exporter	0	87.41%
	TCDB	3.A.1.210.2	Metal exporter	0	96.63%
AO090026000828	TCDB	2.A.19.4.5	Sodium/calcium antiporter	5.00E-12	20.00%
	TCDB	2.A.19.4.4	Sodium/potassium/calcium exchanger	2.00E-27	24.83%
AO090038000088	TCDB	3.A.3.1.7	P-type ATPase	0	100.00%
	TCDB	3.A.3.1.7	P-type ATPase	0	100.00%
AO090038000194	TransportDB	2.A.44	This family is currently empty	1.00E-44	97.37%
	TransportDB	2.A.44	This family is currently empty	2.00E-36	99.64%
AO090038000314	TCDB	1.A.11.3.2	Ammonium transporter	9.00E-160	97.49%
	TCDB	1.A.11.3.3	Ammonium transporter	0	88.68%
AO090038000322	TCDB	3.A.3.2.2	P-type ATPase	0	84.96%
	TCDB	3.A.3.2.2	P-type ATPase	0	96.76%
AO090038000399	TCDB	3.A.1.31.1	Possible ABC transporter permease for cobalt	3.00E-23	72.28%
	TCDB	3.A.1.31.1	Possible ABC transporter permease for cobalt	2.00E-23	85.66%
AO090038000421	TransportDB	3.A.1	ATP-binding Cassette	7.00E-111	98.28%
	TransportDB	3.A.1	ATP-binding Cassette	6.00E-108	97.65%
AO090038000467	TCDB	2.A.6.6.6	Niemann-Pick C1-like protein	1.00E-177	98.88%
	TCDB	2.A.6.6.3	Niemann-Pick type C-related protein 1	0	90.77%

Gene name*	Database	TCID	Predicted function**	E-value	%Alignment
AO090102000102	TCDB	2.A.36.4.4	Alkali metal cation efflux porter, Cnh1	0	73.73%
	TCDB	2.A.36.4.4	Alkali metal cation efflux porter, Cnh1	0	89.45%
AO090102001037	TCDB	3.D.2.4.1	Proton-translocating transhydrogenase	5.00E-128	99.78%
	TCDB	3.D.2.3.1	Proton-translocating transhydrogenase	1.00E-158	42.45%
AO090102000567	TCDB	1.C.82.1.1	Pore-forming amphipathic helical peptide family	6.00E-20	65.56%
	TCDB	1.C.82.1.1	Pore-forming amphipathic helical peptide family	4.00E-20	86.75%
AO090102000573	TCDB	2.A.9.1.2	Cytochrome oxidase	2.00E-29	58.67%
	TCDB	2.A.9.1.1	Cytochrome oxidase, Oxa1p	2.00E-37	78.86%
AO090102000593	TransportDB	3.A.5	General secretory pathway family	3.00E-109	96.65%
	TransportDB	3.A.5	General secretory pathway family	1.00E-51	97.91%
AO090102000608	TCDB	9.A.36.1.2	Scramblase	2.00E-58	57.94%
	TCDB	9.A.36.1.2	Scramblase	1.00E-58	78.29%
AO090103000274	TCDB	2.A.22.6.1	Sodium/imino-acid transporter	4.00E-40	66.17%
	TCDB	2.A.22.3.2	Sodium and chloride dependent GABA transporter	1.00E-53	72.12%
	TransportDB	2.A.22	Sodium symporter	1.00E-47	76.44%
	TransportDB	2.A.22	Sodium symporter	2.00E-37	72.97%
AO090103000412	TCDB	2.A.2.1.4	-	3.00E-150	100.00%
	TCDB	2.A.2.1.2	Fucosyl-α-1,6-N-acetylglucosamine uptake porter	2.00E-164	95.94%
AO090120000141	TCDB	1.A.35.5.1	Magnesium transporter	2.00E-78	56.92%
	TCDB	1.A.35.5.1	Magnesium transporter	1.00E-87	70.00%
AO090120000214	TCDB	1.A.56.1.4	Copper transporter	4.00E-33	80.47%
	TCDB	1.A.56.1.4	Copper transporter	3.00E-34	89.21%
AO090120000217	TCDB	2.A.6.6.5	3-hydroxy-3-methylglutaryl (HMG)-CoA reductase	1.00E-166	76.13%
	TCDB	2.A.6.6.5	3-hydroxy-3-methylglutaryl (HMG)-CoA reductase	3.00E-167	49.66%
AO090120000224	TransportDB	9.A.19	Orphan transporter	3.00E-105	91.39%
	TransportDB	9.A.19	Orphan transporter	1.00E-99	84.62%
AO090308000016	TCDB	8.B.9.1.1	Triflin	2.00E-18	78.75%
	TCDB	8.B.9.1.2	Natrin	2.00E-19	57.32%
AO090701000011	TCDB	8.A.15.1.1	Potassium channel-associated protein	5.00E-23	24.71%
	TCDB	8.A.15.1.1	Potassium channel-associated protein	3.00E-23	21.02%
AO090701000344	TCDB	2.A.41.3.1	Nucleoside permease	8.00E-106	84.04%
	TCDB	2.A.41.3.1	Nucleoside permease	5.00E-106	83.00%
AO090701000710	TCDB	3.A.1.208.2	Hepatic canalicular conjugate exporter	0	97.52%
	TCDB	3.A.1.208.11	Metal resistance protein	0	99.14%
AO09M000000001	TCDB	3.A.2.1.3	F-type ATPase	4.00E-11	100.00%
	TCDB	3.A.2.1.3	F-type ATPase	2.00E-11	100.00%
AO09M000000013	TCDB	3.D.1.2.1	NADH dehydrogenase	1.00E-11	79.26%
	TCDB	3.D.1.2.1	NADH dehydrogenase	2.00E-20	87.60%
AO090001000748	TransportDB	2.A.66	polysaccharide export	5.00E-04	30.72%
	TransportDB	2.A.66	polysaccharide export	5.00E-05	26.17%
AO090012000733	TransportDB	2.A.25	sodium ion:alanine symporter	3.00E-17	32.40%
	TransportDB	2.A.25	sodium ion:alanine symporter	8.00E-18	19.87%

Gene name*	Database	TCID	Predicted function**	E-value	%Alignment
AO090038000297	TransportDB	1.A.1	potassium ion channel	6.00E-17	87.45%
	TransportDB	1.A.1	potassium ion channel	1.00E-07	13.88%
AO090011000831	TCDB	2.A.5.5.1	Zinc transporter	6.00E-09	53.70%
	TCDB	2.A.5.5.1	Zinc transporter	5.00E-11	31.71%
AO09M000000014	TCDB	3.D.4.8.1	Cytochrome oxidase	2.00E-91	98.42%
	TCDB	3.D.4.8.1	Cytochrome oxidase	1.00E-91	100.00%
AO09M000000015	TCDB	3.D.1.6.2	NADH-ubiquinone oxidoreductase	2.00E-45	100.00%
	TCDB	3.D.1.6.2	NADH-ubiquinone oxidoreductase	1.00E-45	100.00%
AO09M000000016	TransportDB	2.A.63	Proton antiporter	2.00E-150	99.08%
	TransportDB	2.A.63	Proton antiporter	9.00E-127	95.77%

**Gene name of Candidate transporter gene*

***Predicted transporter functions are based on accession number of TCID which links with protein knowledgebase (UniProtKB).*

Table S2. List of 123 candidate transporter genes with assigned K numbers and KEGG orthology definition

Gene name	GhostKOALA	BlastKOALA	KEGGMapper	KEGG orthology definition
AO090001000091	K05770	K05770	K05770	Benzodiazapine receptor
AO090001000747	K12669	K12669	K12669	Oligosaccharyltransferase complex subunit gamma
AO090001000748	K06316	K06316	K06316	Oligosaccharide translocation protein RFT1
AO090003000050	K02429	K02429	K02429	MFS transporter, FHS family, L-fucose permease
AO090003000443	K03317	K03317	K03317	Concentrative nucleoside transporter, CNT family
AO090003000454	K07936	K07936	K07936	GTP-binding nuclear protein Ran
AO090003000460	K08008	K08008	K08008	NADPH oxidase
AO090003000688	K01551	K01551	K01551	Arsenite-transporting ATPase [EC:3.6.3.16]
AO090003000693	K01381	K01381	K01381	Saccharopepsin [EC:3.4.23.25]
AO090003000798	K14708	K14708	K14708	Solute carrier family 26 (sodium-independent sulfate anion transporter)
AO090003000920	K14430	K14430	K14430	Phosphate transporter
AO090003001119	K12346	K12346	K12346	Metal iron transporter
AO090003001233	K15014	K15014	K15014	Solute carrier family 29 (equilibrative nucleoside transporter)
AO090003001402	K16073	K16073	K16073	Magnesium transporter
AO090003001404	K14640	K14640	K14640	Solute carrier family 20 (sodium-dependent phosphate transporter)
AO090005000019	K03457	K03457	K03457	Nucleobase:cation symporter-1, NCS1 family
AO090005000026	K14709	K14709	K14709	Solute carrier family 39 (zinc transporter), member 1/2/3
AO090005000114	K16261	K16261	K16261	Yeast amino acid transporter
AO090005000455			K03458	Nucleobase:cation symporter-2, NCS2 family
AO090005000775	K12386	K12386	K12386	Cystinosin
AO090005000842	K01535	K01535	K01535	H^+-transporting ATPase [EC:3.6.3.6]
AO090005000980	K03857	K03857	K03857	Phosphatidylinositol glycan, class A [EC:2.4.1.198]
AO090005001175	K13341	K13341	K13341	Peroxin-7
AO090005001300	K15633	K15633	K15633	2,3-bisphosphoglycerate-independent phosphoglycerate mutase [EC:5.4.2.12]
AO090005001332	K03325	K03325	K03325	Arsenite transporter, ACR3 family
AO090005001455	K17800	K17800	K17800	LETM1 and EF-hand domain-containing protein 1, mitochondrial
AO090005001624	K14298	K14298	K14298	mRNA export factor
AO090009000183	K17907	K17907	K17907	Autophagy-related protein 9
AO090009000400	K15275	K15275	K15275	Solute carrier family 35 (UDP-galactose transporter)
AO090009000405	K05863	K05863	K05863	Solute carrier family 25 (mitochondrial adenine nucleotide translocator)
AO090009000636	K12041	K12041	K12041	Solute carrier family 9 (sodium/hydrogen exchanger)
AO090009000637	K15378	K15378	K15378	Solute carrier family 45
AO090009000651	K05658	K05658	K05658	ATP-binding cassette, subfamily B (MDR/TAP) [EC:3.6.3.44]
AO090009000668	K12562	K12562	K12562	Amphiphysin
AO090009000677	K15176	K15176	K15176	RNA polymerase-associated protein CTR9
AO090009000688	K15356	K15356	K15356	GDP-mannose transporter
AO090010000135	K14685	K14685	K14685	Solute carrier family 40 (iron-regulated transporter)
AO090010000212	K01011	K01011	K01011	Thiosulfate/3-mercaptopyruvate sulfurtransferase [EC:2.8.1.1 2.8.1.2]
AO090010000229	K03305	K03305	K03305	Proton-dependent oligopeptide transporter, POT family
AO090010000475	K00411	K00411	K00411	Ubiquinol-cytochrome c reductase iron-sulfur subunit [EC:1.10.2.2]

Gene name	GhostKOALA	BlastKOALA	KEGGMapper	KEGG orthology definition
AO090010000482	K02133	K02133	K02133	F-type H^+-transporting ATPase subunit beta [EC:3.6.3.14]
AO090010000775	K15278	K15278	K15278	Solute carrier family 35 (UDP-xylose/UDP-N-acetylglucosamine transporter)
AO090011000202	K05916	K05916	K05916	Nitric oxide dioxygenase [EC:1.14.12.17]
AO090011000320	K05285	K05285	K05285	Phosphatidylinositol glycan, class N [EC:2.7.-.-]
AO090011000329	K09885	K09885	K09885	Aquaporin rerated protein, other eukaryote
AO090011000638	K04043	K04043	K04043	Molecular chaperone DnaK
AO090011000817	K07241	K07241	K07241	High-affinity nickel-transport protein
AO090011000831	K07238	K07238	K07238	Zinc transporter, ZIP family
AO090012000623	K02575	K02575	K02575	MFS transporter, NNP family, nitrate/nitrite transporter
AO090012000733	K01613	K01613	K01613	Phosphatidylserine decarboxylase [EC:4.1.1.65]
AO090012000773	K14950	K14950	K14950	Cation-transporting ATPase 13A1 [EC:3.6.3.-]
AO090012000797	K02146	K02146	K02146	V-type H^+-transporting ATPase subunit d
AO090012000901	K14640	K14640	K14640	Solute carrier family 20 (sodium-dependent phosphate transporter)
AO090012000997	K08341	K08341	K08341	GABA(A) receptor-associated protein
AO090020000415	K00234	K00234	K00234	Succinate dehydrogenase (ubiquinone) flavoprotein subunit [EC:1.3.5.1]
AO090020000492	K01969	K01969	K01969	3-methylcrotonyl-CoA carboxylase beta subunit [EC:6.4.1.4]
AO090023000569		K05389	K05389	Potassium channel subfamily K, other eukaryote
AO090023000801	K01958	K01958	K01958	Pyruvate carboxylase [EC:6.4.1.1]
AO090023000895	K15040	K15040	K15040	Voltage-dependent anion channel protein 2
AO090026000231	K08794	K08794	K08794	Calcium/calmodulin-dependent protein kinase I [EC:2.7.11.17]
AO090026000255	K03549	K03549	K03549	KUP system potassium uptake protein
AO090026000404	K00624	K00624	K00624	Carnitine O-acetyltransferase [EC:2.3.1.7]
AO090026000432	K05012	K05012	K05012	Chloride channel 3/4/5
AO090026000441	K14715	K14715	K14715	Solute carrier family 39 (zinc transporter)
AO090026000611	K03165	K03165	K03165	DNA topoisomerase III [EC:5.99.1.2]
AO090026000828	K13754	K13754	K13754	Solute carrier family 24 (sodium/potassium/calcium exchanger)
AO090038000088	K01539	K01539	K01541*	Sodium/potassium-transporting ATPase subunit alpha [EC:3.6.3.9]
				* H^+/K^+-exchanging ATPase [EC:3.6.3.10]
AO090038000314	K03320	K03320	K03320	Ammonium transporter, Amt family
AO090038000322	K01537	K01537	K01537	Ca^{2+}-transporting ATPase [EC:3.6.3.8]
AO090038000399	K06174	K06174	K06174	ATP-binding cassette, sub-family E, member 1
AO090038000467	K12385	K12385	K12385	Niemann-Pick C1 protein
AO090102001037	K00323	K00323	K00323	NAD(P) transhydrogenase [EC:1.6.1.2]
AO090102000567	K02863	K02863	K02863	Large subunit ribosomal protein L1
AO090102000573	K03217	K03217	K03217	YidC/Oxa1 family membrane protein insertase
AO090102000593	K03106	K03106	K03106	Signal recognition particle subunit SRP54
AO090103000274	K05034	K05034	K05034	Solute carrier family 6 (neurotransmitter transporter, GABA)
AO090103000412	K17086	K17086	K17086	Transmembrane 9 superfamily member 2/4
AO090120000141	K16075	K16075	K05747*	Magnesium transporter
				*Wiskott-Aldrich syndrome protein
AO090120000214	K14686	K14686	K14686	Solute carrier family 31 (copper transporter)
AO090120000217	K00021	K00021	K00021	Hydroxymethylglutaryl-CoA reductase (NADPH) [EC:1.1.1.34]

Gene name	GhostKOALA	BlastKOALA	KEGGMapper	KEGG orthology definition
AO090120000224	K00088	K00088	K00088	IMP dehydrogenase [EC:1.1.1.205]
AO090701000011	K04706	K04706	K04706	E3 SUMO-protein ligase PIAS1 [EC: 6.3.2.-]
AO09M000000001		K02125	K02125	F-type H^+-transporting ATPase subunit 8
AO09M000000013			K03880	NADH-ubiquinone oxidoreductase chain 3 [EC:1.6.5.3]
AO09M000000014	K02261	K02261	K02261	cytochrome c oxidase subunit 2
AO09M000000015			K03882	NADH-ubiquinone oxidoreductase chain 4L [EC:1.6.5.3]
AO09M000000016	K03883	K03883	K03883	NADH-ubiquinone oxidoreductase chain 5 [EC:1.6.5.3]
AO090003000307				Unclassified process
AO090003000506				Unclassified process
AO090003000854				Unclassified process
AO090003001232				Unclassified process
AO090003001489				Unclassified process
AO090005000714				Unclassified process
AO090005001209				Unclassified process
AO090005001365				Unclassified process
AO090005001573				Unclassified process
AO090009000002				Unclassified process
AO090009000197				Unclassified process
AO090009000582				Unclassified process
AO090009000622				Unclassified process
AO090010000592				Unclassified process
AO090011000535				Unclassified process
AO090011000587				Unclassified process
AO090011000649				Unclassified process
AO090011000912				Unclassified process
AO090012000168				Unclassified process
AO090012000315				Unclassified process
AO090012000787				Unclassified process
AO090012000865				Unclassified process
AO090012001026				Unclassified process
AO090020000192				Unclassified process
AO090020000572				Unclassified process
AO090023000590				Unclassified process
AO090023000892				Unclassified process
AO090026000661				Unclassified process
AO090038000194				Unclassified process
AO090038000297				Unclassified process
AO090038000421				Unclassified process
AO090102000102				Unclassified process
AO090102000608				Unclassified process
AO090308000016				Unclassified process
AO090701000344				Unclassified process

Gene name	GhostKOALA	BlastKOALA	KEGGMapper	KEGG orthology definition
AO090701000710				Unclassified process

Table S3. List of 65 transporter genes and functions involved in *A. oryzae* metabolism

Gene name	Transporter description based on KEGG orthology
AO090005000842	H^+-transporting ATPase [EC:3.6.3.6]
AO090003000050	MFS transporter, FHS family, L-fucose permease
AO090003000443	Concentrative nucleoside transporter, CNT family
AO090003000688	Arsenite-transporting ATPase [EC:3.6.3.16]
AO090003000798	Solute carrier family 26 (sodium-independent sulfate anion transporter)
AO090003000920	Phosphate transporter
AO090003001119	Metal iron transporter
AO090003001233	Solute carrier family 29 (equilibrative nucleoside transporter)
AO090003001402	Magnesium transporter
AO090003001404	Solute carrier family 20 (sodium-dependent phosphate transporter)
AO090005000019	Nucleobase:cation symporter-1, NCS1 family
AO090005000026	Solute carrier family 39 (zinc transporter), member 1/2/3
AO090005000114	Yeast amino acid transporter
AO090005000455	Nucleobase:cation symporter-2, NCS2 family
AO090005001332	Arsenite transporter, ACR3 family
AO090005001455	LETM1 and EF-hand domain-containing protein 1, mitochondrial
AO090009000400	Solute carrier family 35 (UDP-galactose transporter)
AO090009000405	Solute carrier family 25 (mitochondrial adenine nucleotide translocator)
AO090009000636	Solute carrier family 9 (sodium/hydrogen exchanger)
AO090009000637	Solute carrier family 45
AO090009000651	ATP-binding cassette, subfamily B (MDR/TAP) [EC:3.6.3.44]
AO090009000688	GDP-mannose transporter
AO090010000135	Solute carrier family 40 (iron-regulated transporter)
AO090010000212	Thiosulfate/3-mercaptopyruvate sulfurtransferase [EC:2.8.1.1 2.8.1.2]
AO090010000229	Proton-dependent oligopeptide transporter, POT family
AO090010000475	Ubiquinol-cytochrome c reductase iron-sulfur subunit [EC:1.10.2.2]
AO090010000482	F-type H^+-transporting ATPase subunit beta [EC:3.6.3.14]
AO090010000775	Solute carrier family 35 (UDP-xylose/UDP-N-acetylglucosamine transporter)
AO090011000329	Aquaporin rerated protein, other eukaryote
AO090011000817	High-affinity nickel-transport protein
AO090012000623	MFS transporter, NNP family, nitrate/nitrite transporter
AO090012000773	Cation-transporting ATPase 13A1 [EC:3.6.3.-]
AO090012000797	V-type H^+-transporting ATPase subunit d
AO090012000901	Solute carrier family 20 (sodium-dependent phosphate transporter)
AO090023000569	Potassium channel subfamily K, other eukaryote
AO090023000895	Voltage-dependent anion channel protein 2
AO090026000255	KUP system potassium uptake protein
AO090026000432	Chloride channel 3/4/5
AO090026000441	Solute carrier family 39 (zinc transporter)

Gene name	Transporter description based on KEGG orthology
AO090026000828	Solute carrier family 24 (sodium/potassium/calcium exchanger)
AO090038000088	Sodium/potassium-transporting ATPase subunit alpha [EC:3.6.3.9]
AO090038000314	Ammonium transporter, Amt family
AO090038000322	Ca^{2+}-transporting ATPase [EC:3.6.3.8]
AO090038000399	ATP-binding cassette, sub-family E, member 1
AO090102001037	NAD(P) transhydrogenase [EC:1.6.1.2]
AO090103000274	Solute carrier family 6 (neurotransmitter transporter, GABA)
AO090120000141	Magnesium transporter
AO090120000214	Solute carrier family 31 (copper transporter)
AO090120000217	Hydroxymethylglutaryl-CoA reductase (NADPH) [EC:1.1.1.34]
AO09M000000001	F-type H^+-transporting ATPase subunit 8
AO09M000000013	NADH-ubiquinone oxidoreductase chain 3 [EC:1.6.5.3]
AO090001000748	Oligosaccharide translocation protein RFT1
AO090012000733	Phosphatidylserine decarboxylase [EC:4.1.1.65]
AO090011000831	Zinc transporter, ZIP family
AO09M000000014	cytochrome c oxidase subunit 2
AO09M000000015	NADH-ubiquinone oxidoreductase chain 4L [EC:1.6.5.3]
AO09M000000016	NADH-ubiquinone oxidoreductase chain 5 [EC:1.6.5.3]
AO090001000747	Oligosaccharyltransferase complex subunit gamma
AO090023000801	Pyruvate carboxylase [EC:6.4.1.1]
AO090005000980	Phosphatidylinositol glycan, class A [EC:2.4.1.198]
AO090005001300	2,3-bisphosphoglycerate-independent phosphoglycerate mutase [EC:5.4.2.12]
AO090120000224	IMP dehydrogenase [EC:1.1.1.205]
AO090011000320	Phosphatidylinositol glycan, class N [EC:2.7.-.-]
AO090020000415	Succinate dehydrogenase (ubiquinone) flavoprotein subunit [EC:1.3.5.1]
AO090020000492	3-methylcrotonyl-CoA carboxylase beta subunit [EC:6.4.1.4]

Table S4. List of 65 transporter genes with curated functions from integrative databases of TCDB, KEGG, and PFAM

Gene name	TCID	TCID definition	KEGG orthology	PFAM
AO090005000842	3.A.3.3.6	Plasma menbrane ATPase	H^+-transporting ATPase	E1-E2 ATPase
AO090003000050	2.A.1.7.1	L-fucose permease	MFS transporter, L-fucose permease	Major Facilitator Superfamily
AO090003000443	2.A.41.2.7	Proton-nucleoside transporter	Concentrative nucleoside transporter	Na^+ nucleoside transporter
AO090003000688	3.A.19.1.1	Arsenite-translocating ATPase	Arsenite-transporting ATPase	Anion-transporting ATPase
AO090003000798	2.A.53.1.2	Sulfate permease	Na^+ independent sulfate transporter	Sulfate permease family
AO090003000920	2.A.47.2.2	Phosphate transporter	Phosphate transporter	Na^+ sulfate symporter
AO090003001119	2.A.55.1.1	Metal uptake transporter	Metal iron transporter	Divalent metal ion transporter
AO090003001233	2.A.57.3.1	Nucleoside transporter	Equilibrative nucleoside transporter	Nucleoside transporter
AO090003001402	1.A.35	Magnesium transport permease	Magnesium transporter	Mg^{2+} transporter
AO090003001404	2.A.20	Phosphate symporter	Na^+ dependent phosphate transporter	Phosphate transporter family
AO090005000019	2.A.39.3.1	Allantoin permease	Nucleobase:cation symporter	Permease for cytosine/purines, uracil, thiamine, allantoin
AO090005000026	2.A.5.1.1	Zinc uptake transporter	Zinc transporter	Zinc transporter
AO090005000114	2.A.3.10.2	Amino acid permease	Yeast amino acid transporter	Amino acid permease
AO090005000455	2.A.40.5.1	Putative purine permease	Nucleobase:cation symporter	Permease family
AO090005001332	2.A.59.1.1	Arsenite exporter	Arsenite transporter	Na^+/bile acid co-transporters
AO090005001455	2.A.97.1.4	K^+/H^+ antiporter	LETM1	K^+/H^+ antiporter
AO090009000400	2.A.7.11.1	UDP-galactose:UMP antiporter	UDP-galactose transporter	UAA transporter family
AO090009000405	2.A.29.1.3	ADP/ATP translocase	ADP/ATP translocator	Mitochondrial carrier protein
AO090009000636	2.A.36.1.12	Na^+ /H^+ exchanger	Na^+ /H^+ exchanger	Na^+ /H^+ exchanger
AO090009000637	2.A.2.6.1	Alpha-glucoside permease	Solute carrier family 45	MFS/sugar transport protein
AO090009000651	3.A.1.201.11	Multidrug resistance protein	ATP-binding cassette	ABC transporter
AO090009000688	2.A.7.13.2	GDP-mannose transporter	GDP-mannose transporter	Nucleoside-sugar transporters
AO090010000135	2.A.100.1.3	Ferroportin	Iron-regulated transporter	Ferroportin1 (FPN1)
AO090010000212	9.B.102.4.1	Rhodanese domain protein	3-mercaptopyruvate sulfurtransferase	Rhodanese-like domain
AO090010000229	2.A.17.2.2	Peptide transporter	Oligopeptide transporter	POT family
AO090010000475	3.D.3.2.1	Cytochrome b-c	Ubiquinol-cytochrome c reductase	Ubiquinol-cytochrome-c reductase
AO090010000482	3.A.2	V-type ATPase	F-type H^+-transporting ATPase	ATP synthase
AO090010000775	2.A.7.10.2	UDP N-acetylglucosamine antiporter	UDP-xylose/UDP-N-acetylglucosamine transporter	UAA transporter family
AO090011000329	1.A.8.8.8	Aquaporin	Aquaporin	Aquaporin
AO090011000817	2.A.52.1.3	Nickel transporter	High-affinity nickel-transport protein	High-affinity nickel-transporter
AO090012000623	2.A.1.8.5	Nitrate transporter	Nitrate/nitrite transporter	Major Facilitator Superfamily
AO090012000773	3.A.3.10.1	P-type ATPase	Cation-transporting ATPase	Halo acid dehalogenase
AO090012000797	3.A.2.2.3	V-type ATPase	V-type H^+-transporting ATPase	ATP synthase
AO090012000901	2.A.20.2.2	Phosphate transporter	Na^+ dependent phosphate transporter	Phosphate transporter family
AO090023000569	1.A.1.7.1	Potassium channel	Potassium channel	Ion channel
AO090023000895	1.B.8.1.1	Voltage-dependent anion channel	Voltage-dependent anion channel	Eukaryotic porin
AO090026000255	2.A.72.3.2	Potassium transporter	Potassium uptake transporter	Potassium transporter

Gene name	TCID	TCID definition	KEGG orthology	PFAM
AO090026000432	2.A.49.1.3	Chloride channel	Chloride channel	Voltage gated chloride channel
AO090026000441	2.A.5.7.1	Zinc transporter	Zinc transporter	Zinc transporter
AO090026000828	2.A.19.4.4	Na^+ /K^+/Ca^{2+} exchanger	Na^+ /K^+/Ca^{2+} exchanger	Na^+ /K^+/Ca^{2+} exchanger
AO090038000088	3.A.3.1.7	P-type ATPase	Na^+ /K^+-transporting ATPase	E1-E2 ATPase
AO090038000314	1.A.11.3.2	Ammonium transporter	Ammonium transporter	Ammonium transporter
AO090038000322	3.A.3.2.2	P-type ATPase	Ca^{2+}-transporting ATPase	Cation transporting ATPase
AO090038000399	3.A.1.31.1	Cobalt ABC transporter	ATP-binding cassette	ABC transporter
AO090102001037	3.D.2.4.1	NAD(P) transhydrogenase	NAD(P) transhydrogenase	NAD(P) transhydrogenase
AO090103000274	2.A.22.3.2	Na^+/Cl^- GABA transporter	GABA transporter	Na^+ neurotransmitter symporter
AO090120000141	1.A.35.5.1	Magnesium transporter	Magnesium transporter	Magnesium transporter
AO090120000214	1.A.56.1.4	Copper transporter	Copper transporter	Copper transporter
AO090120000217	2.A.6.6.5	3-hydroxy-3-methylglutaryl (HMG)-CoA reductase	Hydroxymethylglutaryl-CoA reductase (NADPH)	Hydroxymethylglutaryl-coenzyme A reductase
AO09M000000001	3.A.2.1.3	F-type ATPase	F-type H^+-transporting ATPase	Fungal ATP synthase
AO09M000000013	3.D.1.2.1	NADH dehydrogenase	NADH-ubiquinone oxidoreductase	NADH-ubiquinone oxidoreductase
AO090001000748	2.A.66	Polysaccharide exporter	Oligosaccharide translocation protein	Oligosaccharide translocator
AO090012000733	2.A.25	Na^+ alanine symporter	Phosphatidylserine decarboxylase	Phosphatidylserine decarboxylase
AO090011000831	2.A.5.5.1	Zinc transporter	Zinc transporter	Zinc transporter
AO09M000000014	3.D.4.8.1	Cytochrome C oxidase	Cytochrome C oxidase	Cytochrome C oxidase
AO09M000000015	3.D.1.6.2	NADH-ubiquinone oxidoreductase	NADH-ubiquinone oxidoreductase	NADH-ubiquinone oxidoreductase
AO09M000000016	2.A.63	Proton antiporter	NADH-ubiquinone oxidoreductase	Proton-conducting transporter
AO090001000747	9.A.45.1.1	Magnesium transporter	Oligosaccharyltransferase	OST3 / OST6 family
AO090023000801	3.B.1.1.1	Oxaloacetate decarboxylase	Pyruvate carboxylase	Carbamoyl-phosphate synthase
AO090005000980	2.A.38.4.5	Na^+ /K^+ transporter	Phosphatidylinositol glycan	GPI anchor
AO090005001300	2.A.7.22.2	4-amino-4-deoxy-L-arabinose-phosphoundecaprenol flippase	2,3-bisphosphoglycerate-independent phosphoglycerate mutase	BPG-independent PGAM
AO090120000224	9.A.19	Orphan transporter	IMP dehydrogenase	IMP dehydrogenase
AO090011000320	9.A.6.1.1	ATP release protein	Phosphatidylinositol glycan	Phosphatidylinositolglycan
AO090020000415	5.A.4.1.1	This family is currently empty	Succinate dehydrogenase	FAD binding domain
AO090020000492	3.B.1.1.5	Methylmalonyl-CoA decarboxylase	3-methylcrotonyl-CoA carboxylase	Carboxyl transferase domain

Table S5. List of top 10 identified templates for *A. oryzae* H^+-ATPase encoded by AO090005000842 gene

Template	Sequence Identity	Method	Resolution	Sequence Similarity	Coverage	Description
1mhs.1.A	77.47	2DX	8.00Å	0.53	0.94	Plasma Membrane ATPase
3b8c.1.A	40.93	X-ray	3.60Å	0.40	0.82	ATPase 2, plasma membrane-type
4hyt.1.A	24.24	X-ray	3.40Å	0.32	0.87	Sodium/potassium-transporting ATPase subunit alpha-1
3b8e.1.A	24.24	X-ray	3.50Å	0.32	0.87	Sodium/potassium-transporting ATPase subunit alpha-1
3a3y.1.A	23.39	X-ray	2.80Å	0.32	0.87	Na, K-ATPase alpha subunit
2yn9.1.A	24.06	2DX	8.00Å	0.32	0.87	Potassium-transporting ATPase alpha
2c88.1.A	25.69	X-ray	3.10Å	0.32	0.85	Sarcoplasmic/endoplasmic reticulum calcium ATPase 1
3ba6.1.A	25.69	X-ray	2.80Å	0.32	0.85	Sarcoplasmic/endoplasmic reticulum calcium ATPase 1
2c9m.1.A	25.69	X-ray	3.00Å	0.32	0.85	Sarcoplasmic/endoplasmic reticulum calcium ATPase 1
2oa0.1.A	25.69	X-ray	3.40Å	0.32	0.85	Sarcoplasmic/endoplasmic reticulum calcium ATPase 1

Table S6. List of conserved catalytic domains between the *A. oryzae* and *N. crassa* H^+-ATPases with percentage of identity

	N. crassa **H^+-ATPase**		***A. oryzae*** **H^+-ATPase**		**Multiple alignment***	
	**Region**	**Residue number**	**Region**	**Residue number**	**Identical residue**	**Percent identity**
N-terminal extension			r1-46	46	0	0.00
**Activator (A)-domain total**		**220**		**184**	**118**	**64.13**
A-domain (1)	r1-110	110	r47-121	75	43	55.13
A-domain (2)	r175-284	110	r186-294	109	75	68.18
**Phosphorylation (P)-domain total**		**165**		**165**	**152**	**92.12**
P-domain (1)	r357-386	30	r367-396	30	28	93.33
P-domain (2)	r535-669	135	r545-679	135	124	91.85
**Nucleotide-binding (N)-domain total**	**r387-534**	**148**	**r397-544**	**148**	**107**	**72.30**
**Transmembrane (M)-domain total**		**349**		**349**	**281**	**80.52**
M-domain (1)	r111-174	64	r122-185	64	52	81.25
M-domain (2)	r285-356	72	r295-366	72	63	87.50
M-domain (3)	r670-884	213	r680-892	213	166	77.93
**Regulatory (R)-domain total**	**r885-920**	**38**	**r893-930**	**38**	**23**	**60.53**
C-terminal extension			r931-943	13	0	0.00
**Total conserved region**		**920**		**943**	**681**	**76.69**

**CLUSTALW was run on NPS@server*

Table S7. List of average RMSD over the equilibrium of five principal regions in *A. oryzae* and *N. crassa* H^+-ATPases

**Region**	***Aspergillus oryzae*** **H^+-ATPase**	***Neurospora crassa*** **H^+-ATPase**
Activator (A)-domain	1.14±0.034	1.05±0.038
Phosphorylation (P)-domain	0.48±0.011	0.44±0.015
Nucleotide-binding (N)-domain	0.59±0.012	0.71±0.047
Transmembrane (M)-domain	0.71±0.013	0.73±0.017
Regulatory (R)-domain	0.91±0.031	0.73±0.080
Global structure	1.11±0.031	1.14±0.036

Table S8. Comparable RMSF between *A. oryzae* and *N. crassa* H^+-ATPases

***Neurospora crassa* H^+-ATPase**		***Aspergillus oryzae* H^+-ATPase**	
**Residue**	**RMSF**	**Residue**	**RMSF**
VAL37	0.2736	VAL47	0.8555
GLU38	0.269	GLU48	0.8357
ASP39	0.2522	ASP49	0.7861
ASP40	0.2183	GLY50	0.7471
GLU41	0.1851	GLU51	0.7041
ASP42	0.1806	ASP52	0.6627
-		VAL53	0.6246
GLU43	0.1926	GLU54	0.7187
ASP44	0.1801	ASN55	0.6676
ILE45	0.2241	ILE56	0.6071
ASP46	0.2872	ASP57	0.5688
ALA47	0.2808	THR58	0.549
LEU48	0.2431	LEU59	0.4859
ILE49	0.29	ILE60	0.3621
GLU50	0.3604	GLU61	0.3617
ASP51	0.4191	GLU62	0.26
LEU52	0.5476	LEU63	0.3145
GLU53	0.5065	GLU64	0.2732
SER54	0.5867	SER65	0.277
HIS55	0.601	LEU66	0.264
ASP56	0.6147	ASP67	0.2633
-		GLY68	0.3786
-		ASN69	0.4
-		LEU70	0.482
GLY57	0.6798	ASP71	0.4741
HIS58	0.6456	TYR72	0.4707
ASP59	0.6938	ASN73	0.4463
ALA60	0.8036	ASP74	0.3927
GLU61	0.8563	SER75	0.4165
GLU62	0.9386	GLY76	0.4685
GLU63	0.9314	GLN77	0.5618
GLU64	1.0766	ASP78	0.5634
GLU65	1.0101	GLU79	0.4859
GLU66	0.9412	LYS80	0.4685
ALA67	0.9052	SER81	0.4459
THR68	0.7484	THR82	0.4278
PRO69	0.7231	PRO83	0.4374
GLY70	0.6463	-	
GLY71	0.5547	-	

GLY72	0.492	-	
ARG73	0.4782	CYS84	0.4418
VAL74	0.4066	CYS85	0.4677

***Neurospora crassa* H^+-ATPase**		***Aspergillus oryzae* H^+-ATPase**	
**Residue**	**RMSF**	**Residue**	**RMSF**
VAL75	0.4131	ILE86	0.4439
PRO76	0.3607	SER87	0.392
GLU77	0.3832	GLU88	0.2747
ASP78	0.4007	GLU89	0.2925
MET79	0.3476	LEU90	0.282
LEU80	0.292	LEU91	0.2689
GLN81	0.3238	GLN92	0.2297
THR82	0.2769	THR93	0.2529
ASP83	0.3176	ASP94	0.2419
THR84	0.2667	PRO95	0.2217
ARG85	0.2971	SER96	0.2407
VAL86	0.2311	THR97	0.2843
GLY87	0.3209	GLY98	0.3702
LEU88	0.2546	LEU99	0.2503
THR89	0.256	THR100	0.2797
SER90	0.2497	GLU101	0.2797
GLU91	0.2642	PRO102	0.292
GLU92	0.2261	GLU103	0.2495
VAL93	0.1958	VAL104	0.2104
VAL94	0.2283	LEU105	0.2229
GLN95	0.234	LEU106	0.2358
ARG96	0.1894	ARG107	0.2039
ARG97	0.2076	ARG108	0.1763
ARG98	0.2606	LYS109	0.199
LYS99	0.2258	LYS110	0.2232
TYR100	0.1871	TYR111	0.1919
GLY101	0.2146	GLY112	0.1764
LEU102	0.2162	LEU113	0.176
ASN103	0.2187	ASN114	0.2267
GLN104	0.2057	GLN115	0.2311
MET105	0.2107	MET116	0.2022
LYS106	0.1663	LYS117	0.1914
GLU107	0.1371	GLU118	0.2013
GLU108	0.1245	GLU119	0.2382
LYS109	0.1469	LYS120	0.2499
GLU110	0.1394	GLU121	0.2199
ASN111	0.1221	ASN122	0.1833
HIS112	0.1482	LEU123	0.1807

PHE113	0.1163	ILE124	0.1734
LEU114	0.1454	LEU125	0.1802
LYS115	0.1622	LYS126	0.2298
PHE116	0.1448	PHE127	0.2062
LEU117	0.1331	LEU128	0.1599
GLY118	0.1497	SER129	0.2172
***Neurospora crassa* H^+-ATPase**		***Aspergillus oryzae* H^+-ATPase**	
**Residue**	**RMSF**	**Residue**	**RMSF**
PHE119	0.1707	TYR130	0.247
PHE120	0.1672	PHE131	0.1817
VAL121	0.1696	VAL132	0.2236
GLY122	0.1901	GLY133	0.3045
PRO123	0.1937	PRO134	0.309
ILE124	0.1772	VAL135	0.1996
GLN125	0.183	GLN136	0.2279
PHE126	0.2085	PHE137	0.2612
VAL127	0.2066	VAL138	0.2311
MET128	0.1845	MET139	0.215
GLU129	0.2018	GLU140	0.2511
GLY130	0.2256	ALA141	0.2485
ALA131	0.2125	ALA142	0.2564
ALA132	0.2036	ALA143	0.3212
VAL133	0.2436	ILE144	0.414
LEU134	0.3206	LEU145	0.4067
ALA135	0.5035	ALA146	0.4637
ALA136	0.488	ALA147	0.4634
GLY137	0.5238	GLY148	0.3983
LEU138	0.7222	LEU149	0.2729
GLU139	0.8428	GLN150	0.3147
ASP140	0.7469	ASP151	0.2767
TRP141	0.6256	TRP152	0.2625
VAL142	0.3862	VAL153	0.244
ASP143	0.3282	ASP154	0.2228
PHE144	0.2069	PHE155	0.211
GLY145	0.1781	GLY156	0.2183
VAL146	0.149	VAL157	0.2024
ILE147	0.1432	ILE158	0.1904
CYS148	0.1616	CYS159	0.2175
GLY149	0.1584	ALA160	0.2212
LEU150	0.1334	LEU161	0.2313
LEU151	0.1424	LEU162	0.2565
LEU152	0.157	ILE163	0.3139
LEU153	0.1405	LEU164	0.3409

ASN154	0.1261	ASN165	0.3106
ALA155	0.188	ALA166	0.2919
VAL156	0.2495	SER167	0.3832
VAL157	0.2193	VAL168	0.4257
GLY158	0.1529	GLY169	0.3647
PHE159	0.1511	PHE170	0.2033
VAL160	0.1455	ILE171	0.1877
GLN161	0.1315	GLN172	0.1833
GLU162	0.1178	GLU173	0.1796

Neurospora crassa **H^+-ATPase**		***Aspergillus oryzae*** **H^+-ATPase**	
**Residue**	**RMSF**	**Residue**	**RMSF**
PHE163	0.1586	PHE174	0.1495
GLN164	0.1481	GLN175	0.1761
ALA165	0.187	ALA176	0.1885
GLY166	0.1799	GLY177	0.3203
SER167	0.2251	SER178	0.3454
ILE168	0.1737	ILE179	0.2502
VAL169	0.3292	VAL180	0.2851
ASP170	0.2484	ASP181	0.199
GLU171	0.2029	GLU182	0.1976
LEU172	0.1914	LEU183	0.1796
LYS173	0.1649	LYS184	0.1452
LYS174	0.1524	LYS185	0.1456
THR175	0.1354	THR186	0.1481
LEU176	0.1327	LEU187	0.149
ALA177	0.1142	ALA188	0.1553
LEU178	0.116	LEU189	0.1373
LYS179	0.1132	LYS190	0.1354
ALA180	0.107	ALA191	0.1343
VAL181	0.1057	VAL192	0.1329
VAL182	0.1123	VAL193	0.1482
LEU183	0.1213	LEU194	0.1563
ARG184	0.1669	ARG195	0.1822
ASP185	0.2135	ASN196	0.2148
GLY186	0.2045	SER197	0.1883
THR187	0.1577	HIS198	0.1612
LEU188	0.1295	LEU199	0.133
LYS189	0.1344	ALA200	0.1375
GLU190	0.1307	GLU201	0.1272
ILE191	0.1243	VAL202	0.141
GLU192	0.1146	ASP203	0.1492
ALA193	0.1151	ALA204	0.1894
PRO194	0.1429	SER205	0.2046

GLU195	0.1402	ASP206	0.2043
VAL196	0.1246	VAL207	0.1901
VAL197	0.1452	VAL208	0.1841
PRO198	0.1328	PRO209	0.1784
GLY199	0.135	GLY210	0.183
ASP200	0.1543	ASP211	0.177
ILE201	0.1215	VAL212	0.1521
LEU202	0.0992	LEU213	0.143
GLN203	0.0955	GLU214	0.1451
VAL204	0.0898	ILE215	0.1429
GLU205	0.0956	GLU216	0.1378
GLU206	0.0934	GLU217	0.1382
***Neurospora crassa* H^+-ATPase**		***Aspergillus oryzae* H^+-ATPase**	
**Residue**	**RMSF**	**Residue**	**RMSF**
GLY207	0.0962	GLY218	0.1421
THR208	0.0852	THR219	0.13
ILE209	0.0906	ILE220	0.126
ILE210	0.0902	ILE221	0.122
PRO211	0.1116	PRO222	0.1328
ALA212	0.1088	ALA223	0.1384
ASP213	0.1061	ASP224	0.1425
GLY214	0.1097	GLY225	0.1536
ARG215	0.1193	ARG226	0.1588
ILE216	0.1247	ILE227	0.153
VAL217	0.1447	LEU228	0.1874
THR218	0.1929	SER229	0.2028
ASP219	0.2222	SER230	0.2248
ASP220	0.1693	SER231	0.2085
ALA221	0.1306	VAL232	0.1913
PHE222	0.1258	-	
LEU223	0.1112	LEU233	0.1555
GLN224	0.1081	GLN234	0.1655
VAL225	0.1017	VAL235	0.1534
ASP226	0.1079	ASP236	0.1716
GLN227	0.1321	GLN237	0.1919
SER228	0.1987	SER238	0.243
ALA229	0.389	GLY239	0.2554
LEU230	0.451	ILE240	0.2112
THR231	0.4475	THR241	0.2904
GLY232	0.4371	GLY242	0.359
GLU233	0.3021	GLU243	0.3683
SER234	0.2096	SER244	0.3219
LEU235	0.1504	LEU245	0.2724

ALA236	0.1318	ALA246	0.2144
VAL237	0.1264	VAL247	0.2048
ASP238	0.1297	ASP248	0.2012
LYS239	0.1261	LYS249	0.1886
HIS240	0.1417	ALA250	0.2102
LYS241	0.1497	ASP251	0.2056
GLY242	0.152	GLY252	0.2093
ASP243	0.1356	ASP253	0.192
GLN244	0.1202	THR254	0.1781
VAL245	0.1111	CYS255	0.1534
PHE246	0.1203	TYR256	0.1591
ALA247	0.1062	SER257	0.1356
SER248	0.0984	SER258	0.14
SER249	0.0971	SER259	0.1395
ALA250	0.0956	ALA260	0.1337
***Neurospora crassa* H^+-ATPase**		***Aspergillus oryzae* H^+-ATPase**	
**Residue**	**RMSF**	**Residue**	**RMSF**
VAL251	0.1074	VAL261	0.1584
LYS252	0.1309	LYS262	0.1872
ARG253	0.12	HIS263	0.1547
GLY254	0.1026	GLY264	0.1461
GLU255	0.1039	HIS265	0.1421
ALA256	0.1006	ALA266	0.1557
PHE257	0.1045	ARG267	0.1376
VAL258	0.1017	LEU268	0.1351
VAL259	0.1123	VAL269	0.1534
ILE260	0.109	VAL270	0.1489
THR261	0.1234	THR271	0.1717
ALA262	0.1208	ALA272	0.1579
THR263	0.1251	THR273	0.1733
GLY264	0.1257	GLY274	0.2151
ASP265	0.1396	ASP275	0.1831
ASN266	0.1291	TYR276	0.228
THR267	0.133	THR277	0.153
PHE268	0.1255	PHE278	0.1635
VAL269	0.1326	VAL279	0.1652
GLY270	0.1529	GLY280	0.1763
ARG271	0.1546	ARG281	0.1731
ALA272	0.2162	ALA282	0.2238
ALA273	0.2994	ALA283	0.2512
ALA274	0.2208	ALA284	0.2528
LEU275	0.1348	LEU285	0.2058
VAL276	0.177	VAL286	0.1927

ASN277	0.145	SER287	0.1973
ALA278	0.17	ALA288	0.1858
ALA279	0.2143	ALA289	0.1853
SER280	0.2324	THR290	0.1865
GLY281	0.2133	SER291	0.2499
GLY282	0.2774	GLY292	0.2691
SER283	0.2231	THR293	0.2479
GLY284	0.2227	GLY294	0.231
HIS285	0.148	HIS295	0.2034
PHE286	0.1211	PHE296	0.1763
THR287	0.1157	THR297	0.1779
GLU288	0.1169	GLU298	0.1774
VAL289	0.1079	VAL299	0.1584
LEU290	0.1055	LEU300	0.1517
ASN291	0.1141	ASN301	0.1805
GLY292	0.1159	GLY302	0.1588
ILE293	0.1057	ILE303	0.1328
GLY294	0.101	SER304	0.1314

***Neurospora crassa* H^+-ATPase**		***Aspergillus oryzae* H^+-ATPase**	
**Residue**	**RMSF**	**Residue**	**RMSF**
THR295	0.1099	ILE305	0.1294
ILE296	0.1128	VAL306	0.1133
LEU297	0.0974	LEU307	0.1461
LEU298	0.0979	LEU308	0.1798
ILE299	0.1089	VAL309	0.1794
LEU300	0.1063	LEU310	0.1662
VAL301	0.0968	VAL311	0.1948
ILE302	0.1071	ILE312	0.2592
PHE303	0.1228	MET313	0.1893
THR304	0.1177	THR314	0.1285
LEU305	0.1129	LEU315	0.1445
LEU306	0.1352	LEU316	0.1622
ILE307	0.1496	VAL317	0.1586
VAL308	0.1439	VAL318	0.1774
TRP309	0.1515	TRP319	0.2136
VAL310	0.1796	VAL320	0.2231
SER311	0.2003	SER321	0.2227
SER312	0.2465	SER322	0.2836
PHE313	0.2343	PHE323	0.2624
TYR314	0.1824	TYR324	0.2947
ARG315	0.1791	ARG325	0.2823
SER316	0.1819	SER326	0.3274
ASN317	0.1648	ASN327	0.3013

PRO318	0.1879	GLY328	0.3096
ILE319	0.1882	ILE329	0.2832
VAL320	0.1835	VAL330	0.22
GLN321	0.1589	THR331	0.1787
ILE322	0.1455	ILE332	0.1776
LEU323	0.1533	LEU333	0.1735
GLU324	0.1454	GLU334	0.1471
PHE325	0.1223	PHE335	0.1322
THR326	0.1216	THR336	0.1457
LEU327	0.1305	LEU337	0.1309
ALA328	0.1184	ALA338	0.1042
ILE329	0.1032	ILE339	0.1148
THR330	0.1211	THR340	0.1333
ILE331	0.1339	MET341	0.1236
ILE332	0.121	ILE342	0.0985
GLY333	0.0927	GLY343	0.0913
VAL334	0.0918	VAL344	0.0995
PRO335	0.0966	PRO345	0.1042
VAL336	0.1146	VAL346	0.1197
GLY337	0.1135	GLY347	0.1249
LEU338	0.1055	LEU348	0.1375
***Neurospora crassa* H^+-ATPase**		***Aspergillus oryzae* H^+-ATPase**	
**Residue**	**RMSF**	**Residue**	**RMSF**
PRO339	0.1086	PRO349	0.1294
ALA340	0.0967	ALA350	0.1253
VAL341	0.0912	VAL351	0.135
VAL342	0.104	VAL352	0.1393
THR343	0.1155	THR353	0.1487
THR344	0.1258	THR354	0.1436
THR345	0.0975	THR355	0.1525
MET346	0.0909	MET356	0.1609
ALA347	0.117	ALA357	0.1448
VAL348	0.1172	VAL358	0.1232
GLY349	0.095	GLY359	0.1275
ALA350	0.1024	ALA360	0.1449
ALA351	0.1167	ALA361	0.1366
TYR352	0.1044	TYR362	0.1158
LEU353	0.0923	LEU363	0.1186
ALA354	0.1123	ALA364	0.1388
LYS355	0.1289	LYS365	0.1385
LYS356	0.1183	LYS366	0.1278
LYS357	0.1132	LYS367	0.1403
ALA358	0.1007	ALA368	0.128

ILE359	0.0969	ILE369	0.1334
VAL360	0.0956	VAL370	0.1367
GLN361	0.0983	GLN371	0.1586
LYS362	0.096	ARG372	0.2018
LEU363	0.1026	LEU373	0.2059
SER364	0.112	SER374	0.2127
ALA365	0.0993	ALA375	0.19
ILE366	0.0955	ILE376	0.1814
GLU367	0.0948	GLU377	0.1576
SER368	0.1013	SER378	0.1299
LEU369	0.0896	LEU379	0.14
ALA370	0.0924	ALA380	0.123
GLY371	0.0978	GLY381	0.1151
VAL372	0.0829	VAL382	0.1004
GLU373	0.0782	GLU383	0.0934
ILE374	0.0753	ILE384	0.0857
LEU375	0.0713	LEU385	0.0793
CYS376	0.0742	CYS386	0.0798
SER377	0.0762	SER387	0.0756
ASP378	0.0735	ASP388	0.0784
LYS379	0.0838	LYS389	0.0826
THR380	0.1049	THR390	0.0896
GLY381	0.1117	GLY391	0.0866
THR382	0.092	THR392	0.0786
***Neurospora crassa* H^+-ATPase**		***Aspergillus oryzae* H^+-ATPase**	
**Residue**	**RMSF**	**Residue**	**RMSF**
LEU383	0.0869	LEU393	0.0821
THR384	0.0975	THR394	0.0953
LYS385	0.1014	ARG395	0.1139
ASN386	0.137	ASN396	0.1142
LYS387	0.1807	LYS397	0.1097
LEU388	0.1905	LEU398	0.1162
SER389	0.1938	SER399	0.1226
LEU390	0.167	LEU400	0.1253
HIS391	0.1253	ALA401	0.1364
ASP392	0.1614	GLU402	0.1386
PRO393	0.11	PRO403	0.1308
TYR394	0.1291	TYR404	0.1497
THR395	0.145	THR405	0.1583
VAL396	0.1864	VAL406	0.1795
ALA397	0.2309	PRO407	0.2087
GLY398	0.2963	GLY408	0.2199
VAL399	0.2957	VAL409	0.1945

ASP400	0.2644	THR410	0.1768
PRO401	0.2074	SER411	0.1944
GLU402	0.1984	GLU412	0.1542
ASP403	0.1809	GLU413	0.1329
LEU404	0.167	LEU414	0.1188
MET405	0.171	MET415	0.1147
LEU406	0.1698	LEU416	0.1193
THR407	0.1566	THR417	0.1127
ALA408	0.1594	ALA418	0.1066
CYS409	0.1441	CYS419	0.1166
LEU410	0.1347	LEU420	0.1209
ALA411	0.1355	ALA421	0.1169
ALA412	0.1471	ALA422	0.1169
SER413	0.1453	SER423	0.1242
ARG414	0.1328	ARG424	0.1106
LYS415	0.1384	LYS425	0.1216
LYS416	0.136	LYS426	0.1104
LYS417	0.129	LYS427	0.1131
GLY418	0.1598	GLY428	0.1214
ILE419	0.152	MET429	0.1253
ASP420	0.1579	ASP430	0.1265
ALA421	0.1657	PRO431	0.1483
ILE422	0.1637	ILE432	0.1197
ASP423	0.1593	ASP433	0.1373
LYS424	0.1794	ARG434	0.1606
ALA425	0.196	ALA435	0.1879
PHE426	0.1937	PHE436	0.1664

***Neurospora crassa* H^+-ATPase**		***Aspergillus oryzae* H^+-ATPase**	
**Residue**	**RMSF**	**Residue**	**RMSF**
LEU427	0.2034	LEU437	0.1505
LYS428	0.2314	ARG438	0.18
SER429	0.2441	ALA439	0.1927
LEU430	0.253	LEU440	0.1742
LYS431	0.2274	LYS441	0.1978
TYR432	0.2306	GLY442	0.1841
TYR433	0.1787	TYR443	0.1567
PRO434	0.1444	PRO444	0.1676
ARG435	0.1764	GLU445	0.1601
ALA436	0.1748	ALA446	0.1579
LYS437	0.1945	LYS447	0.1926
SER438	0.1915	LYS448	0.1762
VAL439	0.1525	ALA449	0.1983
LEU440	0.15	LEU450	0.1798

SER441	0.1384	THR451	0.2039
LYS442	0.1362	GLN452	0.2034
TYR443	0.1361	TYR453	0.1738
LYS444	0.1294	LYS454	0.1854
VAL445	0.122	LYS455	0.1808
LEU446	0.1295	LEU456	0.1953
GLN447	0.1374	GLU457	0.1769
PHE448	0.1401	PHE458	0.1429
HIS449	0.1403	PHE459	0.1223
PRO450	0.1705	PRO460	0.1488
PHE451	0.1327	PHE461	0.1651
ASP452	0.1337	ASP462	0.1584
PRO453	0.1312	PRO463	0.2031
VAL454	0.1388	VAL464	0.1903
SER455	0.1269	SER465	0.1677
LYS456	0.1145	LYS466	0.1546
LYS457	0.133	LYS467	0.1612
VAL458	0.1502	VAL468	0.1409
VAL459	0.1469	THR469	0.1241
ALA460	0.1393	ALA470	0.1174
VAL461	0.1392	VAL471	0.1387
VAL462	0.1361	VAL472	0.1453
GLU463	0.1509	GLN473	0.1756
SER464	0.1648	SER474	0.1876
PRO465	0.1693	PRO475	0.2418
GLN466	0.2347	HIS476	0.2906
GLY467	0.2409	GLY477	0.3141
GLU468	0.2309	GLU478	0.2335
ARG469	0.1983	ARG479	0.1909
ILE470	0.184	ILE480	0.1519
***Neurospora crassa* H^+-ATPase**		***Aspergillus oryzae* H^+-ATPase**	
**Residue**	**RMSF**	**Residue**	**RMSF**
THR471	0.1665	VAL481	0.14
CYS472	0.1466	CYS482	0.1201
VAL473	0.1304	MET483	0.1171
LYS474	0.1146	LYS484	0.1131
GLY475	0.1074	GLY485	0.1406
ALA476	0.0959	ALA486	0.169
PRO477	0.093	PRO487	0.1769
LEU478	0.0935	ILE488	0.2009
PHE479	0.1001	PHE489	0.1964
VAL480	0.112	VAL490	0.169
LEU481	0.1019	LEU491	0.1868

LYS482	0.1182	ASN492	0.2184
THR483	0.1626	THR493	0.2298
VAL484	0.3022	VAL494	0.2253
GLU485	0.3943	LYS495	0.2321
GLU486	0.6005	LYS496	0.2251
ASP487	0.7485	ASP497	0.2549
HIS488	0.7712	HIS498	0.238
PRO489	0.767	PRO499	0.2539
ILE490	0.7964	ILE500	0.2291
PRO491	0.5942	SER501	0.2539
GLU492	0.5062	GLU502	0.2849
GLU493	0.3406	GLY503	0.2644
VAL494	0.1778	VAL504	0.2463
ASP495	0.1724	GLU505	0.2661
GLN496	0.1705	THR506	0.2656
ALA497	0.156	ALA507	0.2292
TYR498	0.1294	TYR508	0.2082
LYS499	0.1243	MET509	0.2326
ASN500	0.1329	SER510	0.2524
LYS501	0.1178	LYS511	0.2361
VAL502	0.0966	VAL512	0.2005
ALA503	0.1095	ALA513	0.2126
GLU504	0.1199	ASP514	0.2495
PHE505	0.1003	PHE515	0.2523
ALA506	0.0899	ALA516	0.2332
THR507	0.1047	VAL517	0.232
ARG508	0.0975	ARG518	0.2934
GLY509	0.0973	GLY519	0.3531
PHE510	0.0841	PHE520	0.2175
ARG511	0.0889	ARG521	0.1786
SER512	0.1041	SER522	0.1577
LEU513	0.0916	LEU523	0.1394
GLY514	0.0986	GLY524	0.1389

***Neurospora crassa* H^+-ATPase**		***Aspergillus oryzae* H^+-ATPase**	
**Residue**	**RMSF**	**Residue**	**RMSF**
VAL515	0.1185	VAL525	0.1234
ALA516	0.1268	ALA526	0.1306
ARG517	0.1441	ARG527	0.1344
LYS518	0.1709	LYS528	0.1519
ARG519	0.1862	CYS529	0.1674
GLY520	0.1939	SER530	0.2147
GLU521	0.252	GLU531	0.2157
GLY522	0.2215	GLY532	0.2173

SER523	0.1721	GLU533	0.1791
TRP524	0.1518	TRP534	0.1964
GLU525	0.1295	GLU535	0.1841
ILE526	0.1298	ILE536	0.1722
LEU527	0.1318	LEU537	0.1478
GLY528	0.1196	GLY538	0.1477
ILE529	0.123	ILE539	0.1361
MET530	0.0944	MET540	0.1258
PRO531	0.0979	PRO541	0.1426
CYS532	0.0915	CYS542	0.1334
MET533	0.1205	SER543	0.143
ASP534	0.1526	ASP544	0.116
PRO535	0.1443	PRO545	0.1241
PRO536	0.1189	PRO546	0.11
ARG537	0.1533	ARG547	0.1088
HIS538	0.1431	HIS548	0.1111
ASP539	0.1148	ASP549	0.116
THR540	0.1024	THR550	0.1104
TYR541	0.1119	ALA551	0.1268
LYS542	0.1117	LYS552	0.1043
THR543	0.0933	THR553	0.0863
VAL544	0.0899	ILE554	0.0907
CYS545	0.0986	HIS555	0.094
GLU546	0.0945	GLU556	0.087
ALA547	0.0867	ALA557	0.0823
LYS548	0.0927	LYS558	0.0927
THR549	0.097	SER559	0.1028
LEU550	0.089	LEU560	0.1016
GLY551	0.0914	GLY561	0.1002
LEU552	0.0856	LEU562	0.0887
SER553	0.0834	SER563	0.0859
ILE554	0.0803	ILE564	0.0829
LYS555	0.0811	LYS565	0.0819
MET556	0.0716	MET566	0.0776
LEU557	0.0676	LEU567	0.0823
THR558	0.0684	THR568	0.0901
***Neurospora crassa* H^+-ATPase**		***Aspergillus oryzae* H^+-ATPase**	
**Residue**	**RMSF**	**Residue**	**RMSF**
GLY559	0.0788	GLY569	0.1082
ASP560	0.0716	ASP570	0.1158
ALA561	0.073	ALA571	0.1203
VAL562	0.0777	VAL572	0.1234
GLY563	0.0868	GLY573	0.1387

ILE564	0.0804	ILE574	0.1285
ALA565	0.0746	ALA575	0.1091
ARG566	0.086	ARG576	0.1178
GLU567	0.0935	GLU577	0.1203
THR568	0.0878	THR578	0.0993
SER569	0.0859	SER579	0.0973
ARG570	0.1031	ARG580	0.1162
GLN571	0.1071	GLN581	0.1092
LEU572	0.0986	LEU582	0.0997
GLY573	0.0941	GLY583	0.1134
LEU574	0.0909	LEU584	0.1081
GLY575	0.119	GLY585	0.2196
THR576	0.1363	THR586	0.381
ASN577	0.1621	ASN587	0.4752
ILE578	0.1863	VAL588	0.462
TYR579	0.2119	TYR589	0.4703
ASN580	0.2098	ASN590	0.4147
ALA581	0.2175	ALA591	0.4224
GLU582	0.2297	GLU592	0.3204
ARG583	0.1808	ARG593	0.2492
LEU584	0.1209	LEU594	0.2053
GLY585	0.1084	GLY595	0.1811
LEU586	0.1232	LEU596	0.1516
GLY587	0.105	GLY597	0.1376
GLY588	0.1219	GLY598	0.1385
GLY589	0.1294	LYS599	0.1345
GLY590	0.0987	GLY600	0.1246
ASP591	0.0922	THR601	0.1371
MET592	0.0993	MET602	0.1487
PRO593	0.1142	PRO603	0.2036
GLY594	0.121	GLY604	0.233
SER595	0.1003	SER605	0.1903
GLU596	0.1142	GLN606	0.1973
VAL597	0.1074	VAL607	0.1628
TYR598	0.1255	TYR608	0.1717
ASP599	0.1249	ASP609	0.1563
PHE600	0.1078	PHE610	0.1381
VAL601	0.1014	VAL611	0.1401
GLU602	0.1016	GLU612	0.1582
***Neurospora crassa* H^+-ATPase**		***Aspergillus oryzae* H^+-ATPase**	
**Residue**	**RMSF**	**Residue**	**RMSF**
ALA603	0.0994	ALA613	0.1183
ALA604	0.0982	ALA614	0.1295

Neurospora crassa H^+-ATPase		*Aspergillus oryzae* H^+-ATPase	
ASP605	0.0898	ASP615	0.105
GLY606	0.0876	GLY616	0.0951
PHE607	0.0771	PHE617	0.0975
ALA608	0.0704	ALA618	0.099
GLU609	0.0778	GLU619	0.1046
VAL610	0.0848	VAL620	0.0967
PHE611	0.0784	PHE621	0.103
PRO612	0.0885	PRO622	0.1004
GLN613	0.0935	GLN623	0.106
HIS614	0.0862	HIS624	0.0982
LYS615	0.0817	LYS625	0.0911
TYR616	0.0904	TYR626	0.0973
ASN617	0.0948	ASN627	0.1016
VAL618	0.0876	VAL628	0.0963
VAL619	0.0886	VAL629	0.0975
GLU620	0.1011	ASP630	0.1101
ILE621	0.103	ILE631	0.1095
LEU622	0.0948	LEU632	0.1038
GLN623	0.0996	GLN633	0.1133
GLN624	0.117	GLN634	0.1261
ARG625	0.1174	ARG635	0.1248
GLY626	0.1099	GLY636	0.127
TYR627	0.096	TYR637	0.108
LEU628	0.0867	LEU638	0.1033
VAL629	0.0819	VAL639	0.0934
ALA630	0.0786	ALA640	0.089
MET631	0.0756	MET641	0.0822
THR632	0.0762	THR642	0.0844
GLY633	0.118	GLY643	0.0953
ASP634	0.1242	ASP644	0.0968
GLY635	0.1262	GLY645	0.1144
VAL636	0.1421	VAL646	0.1063
ASN637	0.1359	ASN647	0.1008
ASP638	0.1242	ASP648	0.0944
ALA639	0.113	ALA649	0.1081
PRO640	0.1065	PRO650	0.1107
SER641	0.0896	SER651	0.0967
LEU642	0.092	LEU652	0.097
LYS643	0.1002	LYS653	0.1148
LYS644	0.0934	LYS654	0.1119
ALA645	0.085	ALA655	0.1019
ASP646	0.0858	ASP656	0.1087

Residue	RMSF	Residue	RMSF
THR647	0.0808	ALA657	0.102
GLY648	0.0792	GLY658	0.1019
ILE649	0.0792	ILE659	0.089
ALA650	0.0817	ALA660	0.0993
VAL651	0.0824	VAL661	0.116
GLU652	0.0953	GLU662	0.1204
GLY653	0.1108	GLY663	0.1083
SER654	0.1426	SER664	0.1239
SER655	0.1397	SER665	0.1396
ASP656	0.1312	ASP666	0.1439
ALA657	0.1279	ALA667	0.1187
ALA658	0.1434	ALA668	0.1085
ARG659	0.1745	ARG669	0.124
SER660	0.1622	THR670	0.1422
ALA661	0.1869	ALA671	0.1322
ALA662	0.0833	ALA672	0.1211
ASP663	0.0866	ASP673	0.1323
ILE664	0.0842	ILE674	0.1136
VAL665	0.0809	VAL675	0.1171
PHE666	0.0792	PHE676	0.1194
LEU667	0.089	LEU677	0.1253
ALA668	0.0906	ALA678	0.1264
PRO669	0.0852	PRO679	0.0909
GLY670	0.0786	GLY680	0.0823
LEU671	0.0805	LEU681	0.0803
GLY672	0.0903	SER682	0.0933
ALA673	0.0897	ALA683	0.0989
ILE674	0.0831	ILE684	0.0943
ILE675	0.0853	ILE685	0.0869
ASP676	0.0905	ASP686	0.0989
ALA677	0.093	ALA687	0.1151
LEU678	0.089	LEU688	0.1138
LYS679	0.0887	LYS689	0.1043
THR680	0.0939	THR690	0.1143
SER681	0.0943	SER691	0.1147
ARG682	0.0885	ARG692	0.0992
GLN683	0.0891	GLN693	0.0969
ILE684	0.0983	ILE694	0.1039
PHE685	0.0938	PHE695	0.0958
HIS686	0.0807	HIS696	0.0871
ARG687	0.0847	ARG697	0.0958
MET688	0.0919	MET698	0.0929

TYR689	0.0858	HIS699	0.082
ALA690	0.0798	ALA700	0.085

***Neurospora crassa* H⁺-ATPase**		***Aspergillus oryzae* H⁺-ATPase**	
**Residue**	**RMSF**	**Residue**	**RMSF**
TYR691	0.0812	TYR701	0.0835
VAL692	0.0869	VAL702	0.0768
VAL693	0.0834	VAL703	0.0752
TYR694	0.0765	TYR704	0.0793
ARG695	0.077	ARG705	0.0737
ILE696	0.0732	ILE706	0.0702
ALA697	0.0778	ALA707	0.0756
LEU698	0.079	LEU708	0.0721
SER699	0.0743	SER709	0.0709
ILE700	0.0761	LEU710	0.079
HIS701	0.0882	HIS711	0.0805
LEU702	0.0808	LEU712	0.0805
GLU703	0.085	GLU713	0.099
ILE704	0.1039	ILE714	0.1247
PHE705	0.1029	PHE715	0.1174
LEU706	0.1078	LEU716	0.1258
GLY707	0.1267	GLY717	0.1587
LEU708	0.1368	LEU718	0.1734
TRP709	0.1367	TRP719	0.1698
ILE710	0.1526	ILE720	0.2118
ALA711	0.174	ALA721	0.2503
ILE712	0.1775	ILE722	0.2561
LEU713	0.1644	MET723	0.2299
ASN714	0.1677	ASN724	0.2464
ARG715	0.1476	GLU725	0.2124
SER716	0.1375	SER726	0.1654
LEU717	0.1201	LEU727	0.1404
ASN718	0.1072	ASN728	0.1611
ILE719	0.1162	ILE729	0.1669
GLU720	0.1178	GLN730	0.1887
LEU721	0.1068	LEU731	0.158
VAL722	0.1083	VAL732	0.1387
VAL723	0.1146	VAL733	0.1442
PHE724	0.1002	PHE734	0.147
ILE725	0.1052	ILE735	0.1483
ALA726	0.1285	ALA736	0.1544
ILE727	0.1179	ILE737	0.1606
PHE728	0.1024	PHE738	0.1609
ALA729	0.1023	ALA739	0.1712

ASP730	0.1118	ASP740	0.153
VAL731	0.1203	ILE741	0.1348
ALA732	0.1138	ALA742	0.1464
THR733	0.1077	THR743	0.162
LEU734	0.1191	LEU744	0.1588
Neurospora crassa **H^+-ATPase**		***Aspergillus oryzae*** **H^+-ATPase**	
**Residue**	**RMSF**	**Residue**	**RMSF**
ALA735	0.2147	ALA745	0.1484
ILE736	0.151	ILE746	0.1446
ALA737	0.1149	ALA747	0.1518
TYR738	0.0964	TYR748	0.1306
ASP739	0.0914	ASP749	0.1238
ASN740	0.0924	LYS750	0.1291
ALA741	0.0941	ALA751	0.1507
PRO742	0.0952	PRO752	0.1236
TYR743	0.0876	TYR753	0.1126
SER744	0.1027	SER754	0.1174
GLN745	0.1048	ARG755	0.1044
THR746	0.1071	THR756	0.107
PRO747	0.1156	PRO757	0.1052
VAL748	0.1098	VAL758	0.1025
LYS749	0.1074	LYS759	0.1036
TRP750	0.1308	TRP760	0.109
ASN751	0.1803	ASN761	0.0986
LEU752	0.2142	LEU762	0.1064
PRO753	0.2627	PRO763	0.1189
LYS754	0.1827	ARG764	0.1263
LEU755	0.13	LEU765	0.1097
TRP756	0.1095	TRP766	0.0934
GLY757	0.1382	GLY767	0.1155
MET758	0.1827	MET768	0.1195
SER759	0.1757	SER769	0.1194
VAL760	0.1042	VAL770	0.1097
LEU761	0.092	LEU771	0.0928
LEU762	0.1003	LEU772	0.1007
GLY763	0.1036	GLY773	0.1094
VAL764	0.091	ILE774	0.0951
VAL765	0.0841	VAL775	0.0849
LEU766	0.0895	LEU776	0.0949
ALA767	0.094	ALA777	0.1005
VAL768	0.0884	ILE778	0.0933
GLY769	0.0918	GLY779	0.099
THR770	0.0986	THR780	0.1148

TRP771	0.097	TRP781	0.1096
ILE772	0.0964	VAL782	0.0964
THR773	0.1084	THR783	0.1028
VAL774	0.1256	LEU784	0.1263
THR775	0.1227	SER785	0.1285
THR776	0.1217	THR786	0.1182
MET777	0.1352	MET787	0.1187
TYR778	0.1532	LEU788	0.1369
Neurospora crassa **H^+-ATPase**		***Aspergillus oryzae*** **H^+-ATPase**	
**Residue**	**RMSF**	**Residue**	**RMSF**
ALA779	0.1851	SER789	0.1422
GLN780	0.2619	GLY790	0.1613
GLY781	0.3584	GLY791	0.199
GLU782	0.391	GLU792	0.2243
ASN783	0.407	GLN793	0.2519
GLY784	0.4103	GLY794	0.3123
GLY785	0.3139	GLY795	0.3526
ILE786	0.2756	ILE796	0.3234
VAL787	0.237	VAL797	0.2806
GLN788	0.1885	GLN798	0.2712
ASN789	0.1699	ASN799	0.3219
PHE790	0.1452	PHE800	0.3155
GLY791	0.1617	GLY801	0.2425
ASN792	0.1419	LYS802	0.1987
MET793	0.1232	ARG803	0.1691
ASP794	0.1138	ASP804	0.1877
GLU795	0.1125	GLU805	0.1939
VAL796	0.1148	VAL806	0.1658
LEU797	0.1039	LEU807	0.1311
PHE798	0.1016	PHE808	0.1401
LEU799	0.1144	LEU809	0.1298
GLN800	0.1145	GLU810	0.1127
ILE801	0.1083	ILE811	0.104
SER802	0.1282	SER812	0.1159
LEU803	0.1108	LEU813	0.1104
THR804	0.0825	THR814	0.0977
GLU805	0.0802	GLU815	0.0989
ASN806	0.0971	ASN816	0.109
TRP807	0.0944	TRP817	0.104
LEU808	0.0837	LEU818	0.095
ILE809	0.0868	ILE819	0.1059
PHE810	0.1024	PHE820	0.1198
ILE811	0.1072	ILE821	0.1124

THR812	0.0935	THR822	0.104
ARG813	0.111	ARG823	0.0928
ALA814	0.1249	ALA824	0.0932
ASN815	0.1081	GLU825	0.0975
GLY816	0.1151	GLY826	0.1115
PRO817	0.1076	PRO827	0.1208
PHE818	0.1032	LEU828	0.1235
TRP819	0.117	TRP829	0.1458
SER820	0.1395	SER830	0.1309
SER821	0.1887	SER831	0.2081
ILE822	0.1284	VAL832	0.2289
***Neurospora crassa* H^+-ATPase**		***Aspergillus oryzae* H^+-ATPase**	
**Residue**	**RMSF**	**Residue**	**RMSF**
PRO823	0.128	PRO833	0.203
SER824	0.1182	SER834	0.1908
TRP825	0.1195	TRP835	0.2005
GLN826	0.0993	GLN836	0.17
LEU827	0.0955	LEU837	0.152
SER828	0.1087	THR838	0.1756
GLY829	0.1055	GLY839	0.1797
ALA830	0.0974	ALA840	0.1521
ILE831	0.1029	ILE841	0.1464
PHE832	0.1138	LEU842	0.169
LEU833	0.1129	VAL843	0.1696
VAL834	0.1058	VAL844	0.1485
ASP835	0.1168	ASP845	0.1759
ILE836	0.13	LEU846	0.2328
LEU837	0.1222	MET847	0.2158
ALA838	0.1191	ALA848	0.1527
THR839	0.1395	THR849	0.1827
CYS840	0.1705	PHE850	0.2213
PHE841	0.1651	PHE851	0.2048
THR842	0.163	CYS852	0.1796
ILE843	0.1896	LEU853	0.1973
TRP844	0.2341	PHE854	0.2163
GLY845	0.2401	GLY855	0.2454
TRP846	0.263	TRP856	0.2296
PHE847	0.2203	PHE857	0.23
GLU848	0.328	VAL858	0.2958
HIS849	0.343	GLY859	0.3197
SER850	0.1853	GLY860	0.3222
ASP851	0.2291	GLN861	0.3524
THR852	0.2075	THR862	0.3742

SER853	0.1788	SER863	0.3793
ILE854	0.1646	ILE864	0.2464
VAL855	0.1726	VAL865	0.2382
ALA856	0.1682	THR866	0.228
VAL857	0.1547	VAL867	0.1761
VAL858	0.1549	VAL868	0.1845
ARG859	0.1557	ARG869	0.1469
ILE860	0.1322	THR870	0.1334
TRP861	0.153	TRP871	0.1809
ILE862	0.1472	VAL872	0.1957
PHE863	0.105	PHE873	0.2175
SER864	0.1138	SER874	0.2179
PHE865	0.1197	ILE875	0.1665
GLY866	0.1165	GLY876	0.1652
***Neurospora crassa* H^+-ATPase**		***Aspergillus oryzae* H^+-ATPase**	
**Residue**	**RMSF**	**Residue**	**RMSF**
ILE867	0.1014	VAL877	0.1776
PHE868	0.0978	PHE878	0.1782
CYS869	0.1107	CYS879	0.1634
ILE870	0.109	VAL880	0.1434
MET871	0.0993	MET881	0.1441
GLY872	0.1089	GLY882	0.198
GLY873	0.1284	GLY883	0.1904
VAL874	0.134	LEU884	0.1334
TYR875	0.1197	TYR885	0.1087
TYR876	0.1178	TYR886	0.1218
ILE877	0.1327	LEU887	0.1254
LEU878	0.1342	LEU888	0.1331
GLN879	0.1451	GLN889	0.1276
ASP880	0.1485	ASP890	0.1393
SER881	0.182	SER891	0.1249
VAL882	0.1936	LYS892	0.1331
GLY883	0.2012	GLY893	0.1617
PHE884	0.1793	PHE894	0.1888
ASP885	0.1433	ASP895	0.1878
ASN886	0.1424	ASN896	0.1919
LEU887	0.1649	ILE897	0.2434
MET888	0.1512	MET898	0.2714
HIS889	0.166	ASN899	0.3058
GLY890	0.1973	GLY900	0.331
LYS891	0.2296	ARG901	0.3551
SER892	0.1941	TRP902	0.3515
PRO893	0.2457	PRO903	0.448

LYS894	0.2239	GLY904	0.476
GLY895	0.2282	SER905	0.4493
ASN896	0.1972	LYS906	0.3403
-		ALA907	0.3317
GLN897	0.1794	SER908	0.3918
LYS898	0.1633	ARG909	0.3634
GLN899	0.1558	GLN910	0.2814
ARG900	0.1507	ARG911	0.3197
SER901	0.1401	GLN912	0.3983
LEU902	0.1305	LYS913	0.3678
GLU903	0.1459	GLU914	0.3132
ASP904	0.1536	ASP915	0.3321
PHE905	0.1342	PHE916	0.2897
VAL906	0.1327	VAL917	0.2908
VAL907	0.1741	VAL918	0.3395
SER908	0.1831	SER919	0.3455
LEU909	0.1578	MET920	0.2751

***Neurospora crassa* H^+-ATPase**		***Aspergillus oryzae* H^+-ATPase**	
**Residue**	**RMSF**	**Residue**	**RMSF**
GLN910	0.1258	GLN921	0.2217
ARG911	0.1348	ARG922	0.2469
VAL912	0.1733	THR923	0.2059
SER913	0.228	SER924	0.1583
THR914	0.2806	THR925	0.1254
GLN915	0.4047	LEU926	0.1619
HIS916	0.4698	HIS927	0.1806
GLU917	0.6357	GLU928	0.2099
LYS918	0.7407	LYS929	0.289
SER919	0.85	SER930	0.4046
